# Human antibody targeting *Vibrio cholerae* O1 O-specific polysaccharide induces an amotile hypovirulent bacterial phenotype: mechanism of protection against cholera

**DOI:** 10.1128/mbio.02235-25

**Published:** 2025-09-12

**Authors:** Smriti Verma, Murat Cetinbas, Meagan Kelly, Stefania Senger, Christina S. Faherty, Jeshina Janardhanan, Chanchal R. Wagh, Taufiqur Rahman Bhuiyan, Fahima Chowdhury, Ashraful Islam Khan, Aklima Akter, Richelle C. Charles, Jason B. Harris, Stephen B. Calderwood, Jens Wrammert, Matthew K. Waldor, Merrill Asp, Jung-Shen Benny Tai, Jing Yan, Peng Xu, Pavol Kováč, Ruslan I. Sadreyev, Firdausi Qadri, Edward T. Ryan

**Affiliations:** 1 Division of Infectious Diseases, Massachusetts General Hospital 2348, Boston, Massachusetts, USA; 2 Division of Pediatric Gastroenterology and Nutrition, Mucosal Immunology and Biology Research Center, Massachusetts General Hospital 2348, Boston, Massachusetts, USA; 3 Department of Molecular Biology, Massachusetts General Hospital 2348, Boston, Massachusetts, USA; 4 Department of Pediatrics, Harvard Medical School 1811 , Boston, Massachusetts, USA; 5 International Centre for Diarrhoeal Disease Research, Bangladesh (ICDDR,B), Dhaka, Bangladesh; 6 Department of Medicine, Harvard Medical School 1811 , Boston, Massachusetts, USA; 7 Department of Immunology and Infectious Diseases, Harvard T. H. Chan School of Public Health 1857 , Boston, Massachusetts, USA; 8 Division of Pediatric Global Health, Massachusetts General Hospital 2348, Boston, Massachusetts, USA; 9 Division of Infectious Disease, Department of Pediatrics, Emory University School of Medicine 12239, Atlanta, Georgia, USA; 10 Emory Vaccine Center, Emory University School of Medicine 12239, Atlanta, Georgia, USA; 11 Department of Microbiology and Immunology, Harvard Medical School 1811 , Boston, Massachusetts, USA; 12 Division of Infectious Diseases, Brigham and Women’s Hospital, Boston, Massachusetts, USA; 13 Howard Hughes Medical Institute, Boston, Massachusetts, USA; 14 Department of Molecular, Cellular and Development Biology, Quantitative Biology Institute, Yale University 5755, New Haven, Connecticut, USA; 15 Laboratory of Bioorganic Chemistry, National Institute of Diabetes and Digestive and Kidney Diseases, National Institutes of Health 2511, Bethesda, Maryland, USA; University of California, Berkeley, Berkeley, California, USA

**Keywords:** *Vibrio cholerae*, mucin, anti-OSP antibody, motility, biofilm, transcriptomic profiling

## Abstract

Antibodies targeting the O-specific polysaccharide (OSP) of *Vibrio cholerae* O1 are crucial determinants of protection against cholera. These antibodies agglutinate bacteria and, even in sub-agglutinating conditions, inhibit *V. cholerae* motility. To explore additional effects of OSP-specific antibodies, we examined the transcriptomic profiles of *V. cholerae* exposed to a human anti-OSP monoclonal antibody in the presence of mucin, the main component of intestinal mucus, and the substance in which *V. cholerae* and mucosal antibodies interact in infected humans. Beyond genes whose transcript levels were affected by either mucin alone or antibody alone, we identified a set of genes whose expression levels were specifically altered in the presence of both anti-OSP antibody and mucin. These genes are involved in diverse processes such as metabolism, transport, stress response, biofilm formation, motility, and second messenger signaling. Additional culture-based assays and a human small intestine enteroid model confirmed the broad impact of OSP-specific antibodies on *V. cholerae*, including the inhibition of motility, downregulation of virulence mechanisms, and a shift of bacterial metabolism toward decreased synthesis of intermediates and precursors in a sessile state secreting extracellular matrix component of a biofilm. Collectively, our findings reveal that antibodies targeting *V. cholerae* OSP markedly transform the pathogen’s physiology and disrupt its virulence program. We propose that these effects explain how antibodies targeting *V. cholerae* OSP mediate protection against cholera at the intestinal surface of infected humans.

**IMPORTANCE:**

Immunity to cholera is largely mediated by antibodies targeting the O-specific polysaccharide (OSP) of *Vibrio cholerae,* including through agglutination as well as inhibition of bacterial motility. Here, we used bacterial transcriptomic, biochemical, and cellular analyses to evaluate additional effects of OSP-specific antibodies on *V. cholerae* in complex media containing mucin and in a human enteroid-derived monolayer colonization model. We found that anti-OSP antibody in mucin impacts bacterial motility, growth, metabolic activity, extracellular matrix production, and levels of cyclic di-GMP. We did not observe a direct effect on bacterial viability, sodium motive force gradient, membrane integrity for large molecules, or virulence gene or regulon expression in bacterial cultures, although cholera toxin detection was significantly decreased in the enteroid model. Our results uncover the broad impact of anti-OSP antibodies in the presence of mucin on *V. cholerae* physiology and suggest several ways OSP-specific antibodies mediate protection against cholera in humans.

## INTRODUCTION

Cholera is a severe, watery diarrheal illness caused by *Vibrio cholerae* O1 or O139 serogroup organisms. Cholera results in millions of cases and tens of thousands of deaths each year in over 50 countries, with the largest burden being borne by children under 5 years of age (1–3). *V. cholerae* can persist in aquatic reservoirs and are typically acquired by ingesting contaminated water or food (4). *V. cholerae* is a human-restricted pathogen. Following ingestion, *V. cholerae* pass to the small intestine; they are highly motile and reach the lower third of villi and the intestinal crypts, where they penetrate the overlying mucus layer (5, 6). Being non-invasive, *V. cholerae* then form micro-aggregate colonies in proximity to intestinal epithelial cells, utilizing several colonization factors such as toxin-coregulated pilus (TCP) (7, 8). *V. cholerae* interacts with host factors such as antibodies in the overlying mucin layer coating the epithelial surface. At the intestinal epithelial surface, *V. cholerae* expresses cholera toxin (CT), which is internalized by the human epithelial cells. CT is an ADP-ribosylating toxin that affects intracellular cyclic AMP, leading to chloride, sodium, and water secretion into the lumen by the affected epithelial cell, resulting in the watery diarrhea characteristic of cholera (9). The expression of CT and TCP by *V. cholerae* is under the control of the ToxR master regulator that recognizes environmental signals in the human intestine (10). Surprisingly, anti-cholera toxin immunity does not provide appreciable protection against cholera (11, 12). Protection against cholera is serogroup-specific, and serogroup specificity is defined by the O-specific polysaccharide (OSP) of the bacterial lipopolysaccharide (LPS). Antibodies against OSP are the main determinants of protection against cholera, but the mechanisms of this protection are uncertain (13–15). Although serum vibriocidal activity correlates with protection against cholera, it appears to be a surrogate marker for yet-to-be-determined activity of OSP-specific antibody active in the lumen of the intestine at the mucosal surface (15–17).

*V. cholerae* is a highly motile organism with a single polar flagellum sheathed with an extension of the outer membrane (thus coated with OSP) (18), and motility-deficient *V. cholerae* are significantly attenuated in colonization (19–21). We have previously cloned OSP-specific antibodies from plasmablasts of humans recovering from cholera in Bangladesh and demonstrated that anti-OSP monoclonal antibodies inhibit *V. cholerae* motility in both agglutinating and subagglutinating conditions (22–24). This effect occurs within 5 min of exposure of *V. cholerae* to OSP-specific antibody, a process that requires a bivalent antibody structure and antibody-mediated cross-linking of OSP molecules (24). Our previous studies have also demonstrated that OSP-specific monoclonal antibody protects against death in mouse models of cholera, inhibiting colonization of the bacteria in intestinal tissue in a motility-dependent manner (24), suggesting motility inhibition to be a prime driver of protection imparted by OSP-specific antibodies. However, other anti-OSP antibodies have been shown to have a wide array of phenotypic effects in *V. cholerae*, *Salmonella* Typhimurium, and *Shigella flexneri* (25–31). These include induction of a bacterial extracellular matrix (25, 26), surface blebbing and disruption of outer membrane integrity (29–31), loss of functionality of the type 3 secretion system (T3SS) in *Salmonella* and *Shigella* (27–29), and a decrease in membrane potential and ATP synthesis (28, 29, 32). Baranova et al. (26) previously evaluated the impact of an anti-*V*. *cholerae* LPS antibody (ZAC-3; directed against the conserved oligosaccharide core/lipid A region of *V. cholerae* O1 LPS) in simple liquid media using transcriptomic profiling. ZAC-3 affected the detection of genes involved in *V. cholerae* energy metabolism, transport, and early stages of biofilm formation (26). The lipid A and core oligosaccharide in *V. cholerae* O1 and O139 are identical, although protection against cholera is serogroup-specific; immunity against O1 does not protect against O139 and *vice versa* (33–35). Since OSP defines serogroup specificity, we were thus interested in more fully defining the impact of OSP-specific antibody (as opposed to anti-oligosaccharide core antibody) on *V. cholerae* beyond the ability of OSP-specific antibody to affect bacterial motility and agglutination. To do this and to assist in down-selecting potential effects, we first assessed the impact of a well-characterized OSP-specific antibody (G1) on *V. cholerae* transcriptomic profiles, then more fully investigated identified pathways and networks. G1 is a high-affinity anti-*V*. *cholerae* O1 OSP monoclonal that recognizes both Inaba and Ogawa OSP serotypes of *V. cholerae* O1 and is expressed as a human IgG1 (22–24).

At the intestinal surface, *V. cholerae* encounter antibodies in a mucus milieu. Mucus comprises 95% water (by weight) held by gel-forming highly glycosylated proteins termed mucins (36). *V. cholerae* interact with mucins via bacterial receptors such as GbpA and RbmC (6, 37, 38) that bind terminal sugars on the mucin proteins and also express enzymes such as TagA (6, 39) and hemagglutinin/protease (HapA) (6, 40, 41) that can degrade mucin, facilitating mucus penetration and possibly providing an alternate energy source for the bacteria at the intestinal surface (39, 41, 42). We therefore performed our analyses in systems using complex media containing mucin to more fully replicate the ecological milieu in which antibody-bacterial interactions would occur in the intestine of infected humans. We also used a human epithelial monolayer infection model to study the impact of OSP-specific antibody on *V. cholerae*-epithelial interactions in a complex human-derived system containing mucus.

## MATERIALS AND METHODS

### Bacterial strains and culture conditions

We used *V. cholerae* El Tor O1 strain C6706, a derivative constitutively expressing red fluorescence protein tdTomato, a rough derivative deficient in perosamine synthase (VC0244::Kan^r^), and a flagellated but nonmotile (VC0893::Kan^r^) strain. For visualizing motility via high-speed live video microscopy, we used C6706 derivative MA042 (*flaA*^A106CS107C^ *flaB*^S106CS107C^ *flaD*^K106CS107C^ ΔVC1807::P_tac_-mScarlet-I, Spec^R^, Δ*cheY3*). Details of all strains used are indicated in Table S1 (5, 43–45). *V. cholerae* were grown in toxin-inducing conditions (TICs) using AKI medium containing sodium bicarbonate (46, 47) without agitation at 37°C for 4 hours as detailed in the Supplemental methods.

### Antibody treatment

We used human monoclonal immunoglobulin G1 (IgG1) targeting *V. cholerae* O1-specific polysaccharide component of LPS (clone G1—CF21.2.G01) and flagellin (clone B12—AT11.1.B12) cloned from patients with cholera in Bangladesh and previously described (22, 23). We have previously characterized these antibodies for attributes, including ability to impact *V. cholerae* motility, agglutination, affinity, specificity, vibriocidal activity, and ability to protect in lethal murine challenge models (22–24). Treatments with antibodies were carried out by diluting TIC bacterial cultures to sub-agglutinating conditions of an optical density at 600 nm (O.D._600_) of ≤0.1 in either Luria Bertani (LB; Sigma), LB with 1% (wt/vol) porcine gastric mucin (LBM; Sigma), M9 minimal medium, tryptone-phosphate broth (48), or culture medium as per experimental requirements. Antibodies were added to a final sub-agglutinating concentration of 0.0125 and/or 0.125 µM (24).

### RNA sequencing library preparation

*V. cholerae* C6706 cultured under TIC were exposed to anti-OSP (G1) and anti-flagellin (B12) in LB or LBM (1%, wt/vol) for 1 hour at room temperature (RT) (26). Bacterial cultures were then pelleted, and total RNA was isolated using lysozyme and RNeasy (Qiagen) per the manufacturer’s instructions. RNA was isolated from cells collected from two independent biological replicates for each condition. The resulting RNA was depleted of ribosomal RNA using a rRNA depletion kit (New England Biolabs), and RNA-seq libraries were constructed using NEBNext Ultra II Directional kit (New England Biolabs). Sequencing was carried out on the Illumina NextSeq 2000 sequencing system in paired-end 50 bp mode.

### RNA-seq analysis

Sequencing reads were mapped to *V. cholerae* O1 biovar El Tor strain N16961 chromosome I and II (49) separately using the Rockhopper package (50). For differential expression analysis, we used EdgeR (51) and classified genes as differentially expressed based on the cutoffs of ±1.5-fold change in expression value and false discovery rate (FDR) below 0.05.

### Metabolism, growth, and viability assessment

The metabolic activity of *V. cholerae* cultured under TIC and diluted to a final O.D._600_ of ≤0.1 in LBM, either exposed or not to G1 or B12 antibodies for 60 min at RT, was assessed using the MTT Assay (52) as described in the Supplemental methods. Growth curves were analyzed by plotting O.D._600_ against time for untreated and antibody-treated *V. cholerae* cultures in LBM until untreated cultures reached an O.D. ~ 0.1 (non-agglutinating conditions [24]), as detailed in the Supplemental methods. The viability of *V. cholerae* upon exposure to G1 or B12 in the presence of mucin was measured using Live/Dead BacLight Bacterial Viability kit (Molecular Probes) following the manufacturer’s instructions and as described in the Supplemental methods.

### Assessment of bacterial membrane integrity

LPS shedding into the culture medium of bacteria exposed to G1 or B12 antibodies in LBM at an O.D. _600_ of ≤0.1 for 60 min at RT was measured using the Limulus Amoebocyte Lysate (LAL) assay (GenScript). The culture medium (supernatant) was also probed for the presence of LPS fragments, membrane protein (zonula occludens toxin, Zot), and intracellular components (RNA polymerase beta subunit) using an enzyme-linked immunosorbent assay (ELISA). The methods are described in detail in Supplemental methods.

### Measurement of membrane electrical potential

Bacterial membrane polarization was measured using the cationic dye JC-1 (Invitrogen) as described by Forbes and colleagues (29) and as detailed in the Supplemental methods.

### Measurement of intracellular sodium

Intracellular sodium concentration was assessed using the fluorescent dye Sodium Green Tetraacetate (Invitrogen) via the protocol modified from Morimoto and colleagues (48) and as described in the Supplemental methods.

### Measurement of ATP

*V. cholerae* were cultured under TIC, diluted in LBM with or without G1 or B12 antibodies to a final O.D._600_ of ≤ 0.1, and incubated at RT for 60 min. ATP concentrations were assayed using the BacTiter-Glo kit (Promega) per the manufacturer’s instructions and as described in the Supplemental methods (29).

### Motility assay

Mucin columns were prepared with LB media containing 1% porcine gastric mucin and 0.3% agarose added to a 1 mL syringe (53, 54). *V. cholerae* cultured under TIC were premixed with G1 or B12 antibodies, loaded onto mucin columns, and allowed to penetrate the media for 3 hours. Fractions of 150 µL were collected from the bottom of the columns, and bacterial numbers were enumerated by diluting samples, plating onto LB-agar plates, and calculating colony-forming units.

### High-speed video microscopy of individual *V. cholerae* in the presence of antibody

*V. cholerae* C6706 strain MA042 was cultured in M9 minimal media (Sigma) supplemented with 2 mM MgSO_4_ (JT Baker), 100 µM CaCl_2_ (JT Baker), and 0.5% glucose at 37°C with shaking for 3.5 hours until an O.D._600_ of 0.8 was reached. Flagella were labeled with Alexa Fluor 488 C5-maleimide (ThermoFisher) at a concentration of 25 µg/mL (diluted in tryptone broth) for 10 min at RT as described in detail in the Supplemental methods. Bacteria were exposed to anti-OSP antibody and imaged using EPI-illumination on a Nikon Ti2-E microscope. Simultaneous dual-color video was achieved with a Cairn OptoSplit II emission image splitter placed at the end of the light path just before the Electron Multiplying CCD, an Andor iXon Life 888 camera.

### Crystal violet assay

Extracellular matrix production by *V. cholerae* was assessed using the dye crystal violet (Electron Microscopy Sciences) as described by Baranova and colleagues (25) and as detailed in the Supplemental methods.

### Cyclic di-GMP measurement

Cyclic bis-(3′-5′)-dimeric guanosine monophosphate (c-di-GMP) level was assayed using a kit (Lucerna, Cyclic-di-GMP assay kit) per the manufacturer’s instructions and as described in the Supplemental methods. This kit has high assay selectivity with a sensitivity of 50 nM of c-di-GMP and a broad dynamic range.

### Colonization of human enteroid-derived polarized epithelial monolayers

Differentiated human epithelial monolayers were generated from enteroids derived from adult terminal ileum and duodenal stem cells, on Transwells (Corning) as described previously (55) and as detailed in the Supplemental methods. *V. cholerae* were cultured in TIC as described above. Following incubation, the O.D._600_ was measured, and bacteria were washed and resuspended in plain DMEM (Gibco) at a density of 1 O.D._600_. Concurrently, 15–20 min before addition of bacteria, G1 or B12 antibodies were added to the apical chambers of the respective Transwells to a final concentration of 0.0125 µM. To probe the impact of mucus, accumulated mucus was washed off in sub-analyses during medium changes and before addition of antibodies and bacteria. *V. cholerae* in DMEM were added to Transwells such that bacteria were diluted 10-fold to a sub-agglutinating final O.D._600_ of ≤0.1 (24, 55). To visualize bacterial colonization, a tdTomato-expressing strain of *V. cholerae* C6706 (Table S1) was overlaid on the enteroid monolayers in the presence and absence of G1 or B12, and plates were incubated at 37°C with 5% CO_2_ for 30 min, fixed with 4% paraformaldehyde, and processed for immunofluorescence as described in the Supplemental methods. Images were analyzed, and the number of bacteria per field was counted. To assess the impact of G1 and B12 on CT secretion, colonized monolayers were incubated at 37°C with 5% CO_2_ for 4 hours, following which the apical supernatant was collected to assess for cholera toxin by ELISA.

### GM1-ELISA for CT

CT levels in monolayer supernatants were assessed using ELISA as described in the Supplemental methods.

### Statistical analysis

Experimental data were compiled and annotated using Microsoft Excel and plotted using Graph Pad Prism version 10. Data are expressed as mean ± standard deviation of at least two to five biological replicates with at least three technical replicates, each as detailed in the figure legends for each experiment. Statistical significance is compared to the absence of antibodies (No Ab) and determined by Student’s *t* test, one-way analysis of variance (ANOVA) with Dunnett’s *post hoc* test for multiple comparisons, or two-way ANOVA with Tukey’s *post hoc* test for multiple comparisons, as applicable. Significance is denoted by asterisks: ****P < 0.0001, ***P < 0.001, **P < 0.01, and *P < 0.05; ns, not significant.

## RESULTS

**Fig 1:**
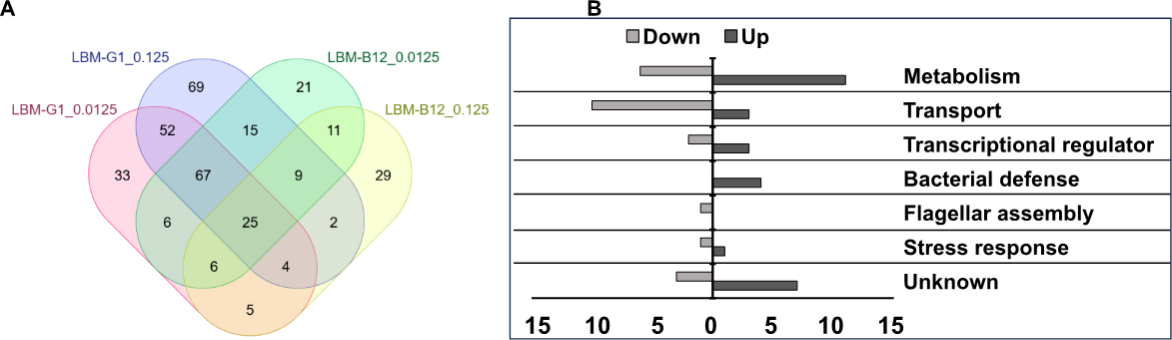
Transcriptomic profiling of *V. cholerae* genes in response to G1 in mucin. (**A**) Venn diagram of the number of *V. cholerae* gene transcripts whose amount was affected in the presence of OSP-specific human monoclonal antibody G1 in mucin vs flagellin-specific antibody B12 in mucin derived from two independent experiments. Transcripts identified to be altered by G1/B12 in mucin were derived by first incorporating the impact of both mucin alone (LB vs LBM) and antibody alone (LB-G1/B12) via Venn Diagram analysis (List S1). Antibodies were used at concentrations of 0.0125 or 0.125 µM. (**B**) Functional groupings of the 52 genes whose expression was altered at both concentrations of LBM-G1 and not in other conditions (*N* = 2).

*V. cholerae* El Tor C6706 was cultured under TICs, followed by exposure to anti-*V*. *cholerae* O1 OSP (G1) monoclonal IgG1 antibody at two concentrations (0.0125 and 0.125 µM) in LB or LBM for 1 hour at RT. For comparison, under the same conditions, *V. cholerae* were also exposed to anti-*V*. *cholerae* flagellin IgG1 monoclonal antibody (B12), which has been previously demonstrated to not impact *V. cholerae* motility (24). Bacterial RNA was subjected to Illumina sequencing. The resulting RNA-seq reads were mapped to the annotated genome of *V. cholerae* El Tor strain N16961 as a reference (49). Differentially expressed genes (DEGs) defined by ± >1.5-fold change in expression and FDR < 0.05 were identified for all treatment groups in comparison to LB (no antibody) as control (List S1): LB vs LBM (also Table S2), LB vs LB-G1 0.0125 µM, LB vs LB-G1 0.125 µM, LB vs LBM-G1 0.0125 µM, LB vs LBM-G1 0.125 µM, LB vs LB-B12 0.0125 µM, LB vs LB-B12 0.125 µM, LB vs LBM-B12 0.0125 µM, and LB vs LBM-B12 0.125 µM. The resulting sets of DEGs were compared between LB vs LBM, LB vs LB-G1/B12, and LB vs LBM-G1/B12 conditions (List S1) to identify genes whose transcript levels were differentially expressed in the presence of antibody in mucin (labeled in Fig. 1A as LBM-G1 or LBM-B12). We then focused on genes that were differentially expressed upon exposure of *V. cholerae* O1 to G1 in mucin and not upon exposure to B12 in mucin (Fig. 1A; Tables S3 to S5; List S1). We analyzed the effect of G1 or B12 antibodies on the expression of select *V. cholerae* genes identified in transcriptional profiling using RT-qPCR (Fig. S1).

### Impact of mucin on *V. cholerae* transcriptome

*V. cholerae* gene expression was significantly impacted by mucin alone (LB vs LBM DEGs; with no antibody [List S1]). Table S2 depicts a partial list of key genes among these DEGs, including genes involved in virulence, such as decreased expression of *toxR* (VC0984)*, tcpH* (VC0827)*, ctxB* (VC1456), several genes encoding TCP biosynthesis proteins, several genes encoding the RTX toxin, as well as genes encoding accessory colonization factor. The largest category affected by the presence of mucin was genes involved in metabolism, such as increased expression of genes involved in metabolism of sialic acid (found abundantly in mucin), including genes involved in scavenging (*nanH*), uptake (*siaPQ*), and catabolism (*nagA* and *nagK*), as well as phosphotransferase system genes. Mucin is a chemoattractant for *V. cholerae* (56), and we detected an impact of mucin on several genes encoding methyl-accepting chemotaxis proteins and chemotaxis proteins (*cheY, cheV, cheB,* and *cheC*) that function as part of the chemosensory system in *V. cholerae*. The expression of most of these genes was increased in the presence of mucin (Table S2). Of three chemosensory systems in *V. cholerae*, one is associated with flagellar motility (57). We found that several genes involved in the biogenesis of the flagellar structure were also affected by the presence of mucin: detection of transcripts of most of the genes encoding the flagellar basal body, hook, and motor was increased along with the flagellin FlaC in the presence of mucin, while detection of transcripts of genes for the primary flagellin, *flaA,* along with *flaD,* was decreased in the presence of mucin (Table S2). Transcripts of genes involved in twitching motility (VC0463, VC0462, and VC1612) were also increased. The expression of many *V. cholerae* transcription factors, two-component phosphorelay proteins, stress response proteins, and signaling cascade proteins was also altered in the presence of mucin (Table S2; List S1). These results highlight a broad impact of mucin on *V. cholerae* physiology.

### Impact of anti-OSP IgG G1 on *V. cholerae* transcriptome in the presence of mucin

*V. cholerae* encounter anti-OSP antibodies in immune or partially immune humans in mucus at the intestinal surface. We thus focused our next efforts on the analysis of *V. cholerae* genes whose transcript levels were altered only in the presence of anti-OSP antibody and mucin (G1 in mucin), and not in mucin alone (LB vs LBM) or following exposure to anti-OSP antibody alone (LB vs LB-G1). The number and category of identified genes by condition and comparison group are shown in Fig. 1. Compared to over 1,200 *V*. *cholerae* genes whose expression was altered when *V. cholerae* was exposed to mucin alone (List S1), we found a smaller number further altered when G1 was added to mucin. A total of 154 *V*. *cholerae* gene transcripts were differentially expressed in response to either of the two concentrations of G1 in the presence of mucin but not in the presence of either concentration of B12 in mucin. Of these, a subset of 52 genes was common in both concentrations, while 69 were found to be affected only by 0.125 µM G1 in mucin and 33 were only impacted by 0.0125 µM G1 in mucin (Fig. 1A; List S1; Tables S3 to S5). These 154 genes belonged to six main functional categories: bacterial stress response, flagellar assembly, bacterial defense, transport, metabolism, and transcriptional regulation (including regulating processes such as motility, biofilm formation, and secondary messenger signaling), along with several genes whose functions are as yet unknown (Fig. 1B; Tables S3 to S5).

Among these 154 genes, we observed decreased expression of VC2138 (*fliS*), a flagellin-specific T3SS chaperone of flagellin monomer (Table 1; Table S3). We also found decreased expression of genes involved in generating the sodium motive force (SMF) required for flagellar function, including VC1016, an ion-translocating oxidoreductase complex subunit B that is a redox-driven ion (Na+) transporter, and VCA0193, encoding a Na+/H+ antiporter (Table 1; Table S3).

**TABLE 1:** Partial list of *V. cholerae* genes identified by transcriptomic profiling whose transcript amounts were altered in the presence of human OSP-specific monoclonal antibody G1 in complex media containing mucin, and not in the presence of human flagellin-specific monoclonal antibody B12 in complex media containing mucin^*a*^

Gene	Annotation	logFC	Product	Associated function
VC0179	*dnvC*	0.589	Dinucleotide cyclase in *Vibrio*	Cyclic nucleotide-based antiphage signaling system
VC0181	*cap3*	0.606	DnvC deubiquitinase	Cyclic nucleotide-based antiphage signaling system
VCA0898	*gnd*	0.619	6-phosphogluconate dehydrogenase	Pentose phosphate pathway/riboflavin synthesis
VC0924	*vpsH*	0.708	Capsular polysaccharide biosynthesis protein CapK	Biofilm formation
VCA0952	*vpsT*	0.8667	LuxR family transcriptional regulator	Biofilm formation
VC1676	*pspC*	1.149	Phage shock protein C	Phage shock response
VCA0681	*V-cGAP1*	1.943	3′3-cGAMP phosphodiesterase	Cyclic di-nucleotide signaling
VC0445	*surA*	−0.592	Survival protein SurA	Envelope stress response
VCA1078	*vqmA*	−0.601	LuxR family transcriptional regulator	Quorum sensing, biofilm formation
VC1016	*rnfB*	−0.642	Ion-translocating oxidoreductase complex subunit B	Redox-driven ion (Na+) transporter
VCA0193		−0.668	Na+/H+ antiporter	Sodium transport
VC2138	*fliS*	−0.823	Chaperone protein FliS	Motility

^*a*^ Genes with increased transcripts are shaded gray. Data are derived from two independent experiments.

The largest cohort of DEGs induced specifically in response to G1 in mucin encoded functions related to metabolism. While exposure of *V. cholerae* to mucin alone increased the expression of genes involved in metabolic pathways, including for alternate energy sources, the addition of G1 to mucin decreased the expression of many metabolic pathways (Fig. 1B; Table S3) such as those involved in the biosynthesis of intermediates including chorismate (VC1507), cysteine (VC1016), coenzyme A (VC0215), as well as genes involved in the transport of molecules, including serine (VC1658), vitamin B12 (VC2381), zinc (VC255), tungstate (VC1524, VC1525), and iron (VC1546). G1 in mucin did increase the expression of VCA0898 encoding phosphogluconate dehydrogenase (*gnd*), an enzyme in the pentose phosphate pathway producing Ribu-5-phosphate, one of the substrates that is required for the biosynthesis of riboflavin; intriguingly, riboflavin produced by *V. cholerae* has been associated with induction of anti-OSP/LPS immune responses in cholera (58, 59). We also observed decreased expression of genes involved in cell wall biosynthesis, including VC2256 (*uppS*) involved in the synthesis of the lipid carrier undecaprenyl phosphate (C55-P) and VC2152 (*dapE*) catalyzing the generation of intermediates involved in the bacterial biosynthesis of lysine and meso-diaminopimelic acid, both being components of the peptidyl moiety of peptidoglycan. These results suggest a broad impact of G1 in mucin on *V. cholerae* metabolism, especially in decreasing its metabolic state.

Adding G1 to mucin also altered the expression of many genes involved in *V. cholerae* stress response, including VC1676 (PspC, phage shock protein C). The phage shock protein (Psp) system is involved in bacterial responses to agents that impact cell membrane function. Expression of the chaperone survival protein A (Sur, VC0445; a member of the σ^E^ cascade) also decreased in response to G1 in mucin. Exposure of *V. cholerae* to G1 in mucin also altered the expression of several genes involved in the cyclic oligonucleotide-based antiphage signaling system, including *dncV* (VC0179) whose gene product preferentially synthesizes the secondary messenger 3′3′-cyclic GMP-AMP (3′3′-cGAMP), as well as VC0681, which is one of three identified phosphodiesterases (designated as V-cGAP1) that catalyze hydrolysis of 3′3′-cGAMP. In the animal commensal strain *Escherichia coli* ECOR31, DnvC regulates biofilm formation and motility (60).

In addition to VC0681, we identified a number of other genes involved in the expression of extracellular matrix and the generation of biofilm in *V. cholerae*. G1 in mucin increased the expression of the transcriptional regulator VpsT encoded by VCA0952, as well as *vpsR* (VC0665). We also detected increased expression of VpsH encoded by VC0924; the *vps* cluster is involved in the synthesis of extracellular matrix by *V. cholerae*. We detected decreased expression of *Vibrio* quorum modulator A (VqmA) encoded by VCA1078, a LuxR-type transcriptional regulator that activates VmqR—a regulatory RNA that suppresses translation of VpsT mRNA (61). G1 in mucin also increased the expression of genes involved in the functioning of the type 2 secretion systems (VC2724, *epsM*), type 6 secretion system (VCA0019, *vasW*), iron metabolism (VC0364, *bfd*, 1), and methylation of 16s rRNA (VC2774).

In summary, our results suggest a broad impact of G1 in mucin on *V. cholerae* metabolism, stress response, biofilm formation, and motility. To explore these findings in more detail, we next undertook more detailed biochemical and functional analyses of identified pathways.

### Impact of anti-OSP IgG G1 and mucin on *V. cholerae* metabolism

**Fig 2:**
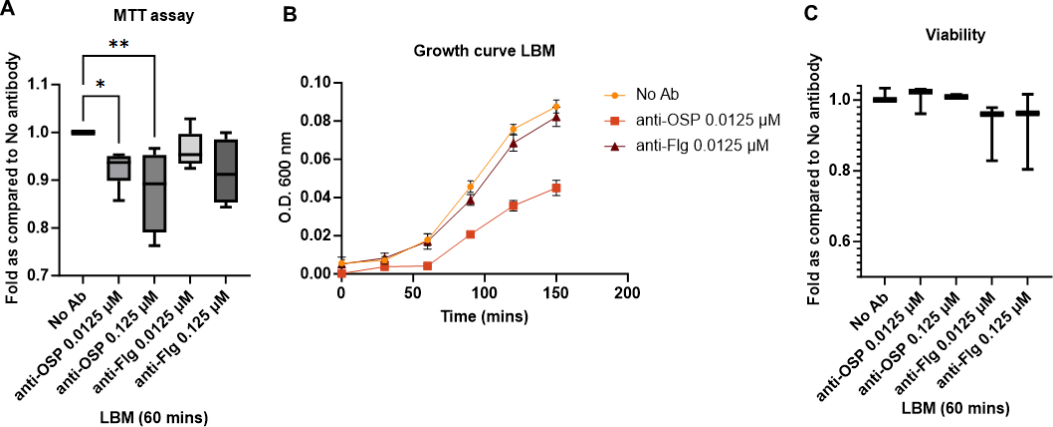
Impact of G1 in mucin on *V. cholerae* metabolism, growth, and viability. (**A**) MTT assay of *V. cholerae* C6706 in the presence or absence of G1 or B12 in mucin. Fold compared to No antibody condition of optical density of dissolved formazan expressed as mean ± standard deviation (sd) of three independent experiments with at least three technical replicates each. Statistical significance compared to the absence of antibodies (No Ab) was determined by two-way analysis of variance with Tukey’s *post hoc* test for multiple comparisons and is denoted by asterisks: *P < 0.05; ** *P* < 0.01. (**B**) Representative growth curve analysis of *V. cholerae* C6706 in the presence or absence of G1 or B12 in mucin expressed as mean ± sd of a representative experiment from three independent experiments with at least three technical replicates each. (**C**) Viability of *V. cholerae* C6706 assessed using the BacLight Live/Dead kit in the presence or absence of G1 or B12 in mucin. Data are expressed as mean ± sd of three biological replicates, each with three technical replicates. Statistical significance compared to the absence of antibodies (No Ab) was determined by one-way ANOVA with Dunnett’s *post hoc* test for multiple comparisons.

As detailed above, the largest category of genes whose expression was altered in response to G1 in mucin encoded for functions relating to bacterial metabolism and transport (Fig. 1B; Table S3). We therefore assessed the metabolic status of *V. cholerae* in response to G1 in mucin by measuring metabolism, growth, viability, levels of ATP generation, as well as membrane potential, the latter two of which have been observed to be disrupted by anti-OSP antibody in simple media in earlier studies (28, 29, 32). Cellular metabolic activity was assessed by comparing the ability to convert tetrazolium dye MTT to formazan. Reduced levels of formazan were observed in the presence of G1 in mucin that were not seen when bacteria were exposed to B12 in mucin, suggesting that one of the effects brought about by G1 in mucin is the slowing of cellular metabolic activity (Fig. 2A). We confirmed this impact (inhibition) on *V. cholerae* growth by OSP-specific antibody in mucin in sub-agglutinating conditions using direct bacterial growth curve analysis (Fig. 2B). We then confirmed that this impact was on growth and metabolism and that OSP-specific antibodies did not directly affect *V. cholerae* viability (Fig. 2C).

**Fig 3:**
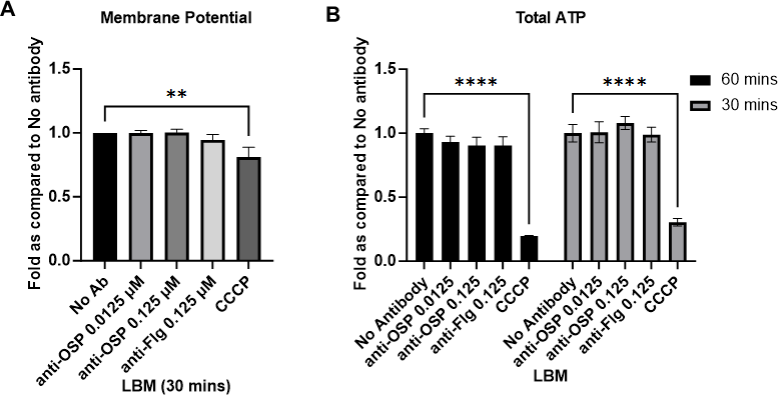
Impact of G1 in mucin on *V. cholerae* membrane potential and ATP levels. (**A**) Membrane potential assessed following exposure to G1 or B12 in mucin for 30 min using potentiometric dye JC-1, followed by calculation of ratio of red fluorescence to green to derive the potential. Fold change in potential as compared to the absence of any antibody (No Ab) was calculated and expressed as mean ± sd of three biological replicates, each with three technical replicates. Statistical significance compared to No Ab was determined by one-way analysis of variance with Dunnett’s *post hoc* test for multiple comparisons and is denoted by asterisks: **P < 0.01. (**B**) Representative data for total ATP levels (combined intracellular and extracellular) measured using the BacTiterGlo Assay kit in the presence or absence of G1 or B12 in mucin. Ionophore CCCP was used as a positive control; see text. Data expressed as mean ± sd fold change compared to the absence of any antibody (No Ab) from five biological replicates, each with three technical replicates. Statistical significance was determined by one-way analysis of variance with Dunnett’s *post hoc* test for multiple comparisons.

Previous literature has suggested that anti-OSP antibodies reduce bacterial membrane potential and ATP generation (28, 29, 32); we thus wanted to explore these aspects as potential contributors to decreased *V. cholerae* growth and metabolism. We assessed membrane potential using the potentiometric dye JC-1. In the presence of high membrane potential, JC-1 aggregates to form structures (J-aggregates) that fluoresce red (Ex: 530 nm and Em: 590 nm) while monomers exhibit green (Ex: 485 nm and Em: 525 nm) fluorescence. We did not detect any membrane depolarization in response to treatment with G1 or B12 in mucin, although a decrease in membrane potential was observed when the ionophore CCCP was used as a control (Fig. 3A). As expected, since ATP synthesis depends on the proton motive force (62), there was no observed reduction in the levels of total ATP (Fig. 3B). Thus, in the presence of G1 in mucin, *V. cholerae* undergo a reduction in metabolic activity and replication that is not associated with the loss of membrane potential and subsequent ATP generation, or due to increased bacterial death.

### Impact of anti-OSP IgG G1 in mucin on *V. cholerae* membrane integrity

**Fig 4:**
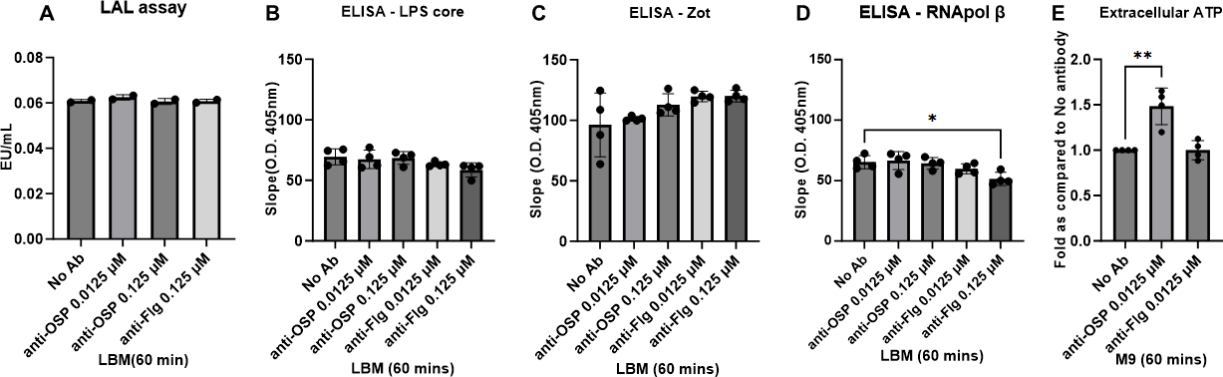
Impact of G1 in mucin on bacterial integrity. (**A**) Representative data for LPS-Lipid A levels in 0.2 µm filtered supernatants of bacteria treated with G1 or B12 in mucin assessed using a LAL assay per the manufacturer’s instructions. Data shown are representative of three biological replicates with two technical replicates each. Statistical significance compared to the absence of antibodies (No Ab) was determined by one-way ANOVA with Dunnett’s *post hoc* test for multiple comparisons. (**B–D**) Cell supernatants of *V. cholerae* C6706 treated with G1 or B12 in mucin were subjected to ELISA with antibodies against core of LPS (**B**), Zot protein (a membrane-associated antigen) (**C**), and RNA polymerase B subunit (an intracellular antigen) (**D**). The data are representative of the results from two independent experiments, each with four replicates. Statistical significance compared to the absence of antibodies (No Ab) was determined by one-way analysis of variance with Dunnett’s *post hoc* test for multiple comparisons and is denoted by asterisks: *P < 0.05. (**E**) ATP released into the culture medium (extracellular ATP) upon exposure of *V. cholerae* to G1 or B12 in minimal medium was measured using the BacTiterGlo assay kit. Data expressed as mean ± sd are composed of three biological replicates, each with three technical replicates. Statistical significance compared to the absence of antibodies (No Ab) was determined by one-way analysis of variance with Dunnett’s *post hoc* test for multiple comparisons and is denoted by asterisks: **P < 0.01.

Several genes whose expression was altered in the presence of G1 in mucin are involved in bacterial defense and envelope stress responses (Table 1; Table S3). Previous studies also suggest that membrane blebbing and outer membrane stress may be a consequence of exposure to anti-LPS antibodies (29–31). We therefore assessed membrane integrity of *V. cholerae* in the presence of G1 in mucin by testing bacterial supernatants for released components of LPS, membrane proteins (zonula occludens toxin [63]), and cytoplasmic contents (RNA polymerase β component and ATP). We did not detect evidence of increased free LPS (Fig. 4A and B) or leakage of membrane-bound and intracellular bacterial proteins (Fig. 4C and D), although we did detect an increase in free ATP in culture supernatants (Fig. 4E), suggesting that although minor leakage/bacterial permeability may occur, no large membrane disruption of *V. cholerae* was evident in the presence of OSP-specific antibody in mucin.

### Impact of anti-OSP IgG G1 on *V. cholerae* C6706 motility, including in medium containing mucin

**Fig 5:**
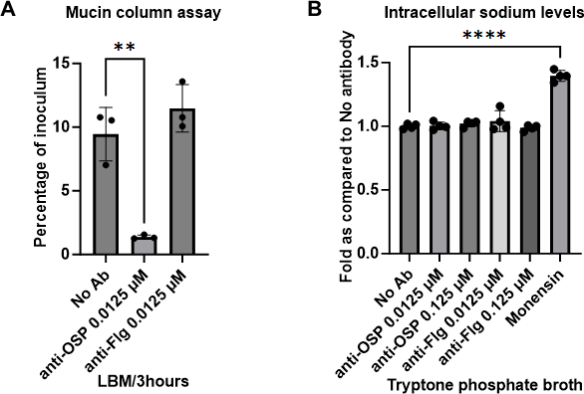
Impact of G1 in mucin on *V. cholerae* motility. (**A**) Mucin motility assay carried out using columns containing 1% (wt/vol) mucin and 0.3% agarose in LB. Data shown are expressed as mean ± sd of a representative of three biological replicates. Statistical significance compared to the absence of antibodies (No Ab) was determined by one-way analysis of variance with Dunnett’s *post hoc* test for multiple comparisons and is denoted by asterisks: **P < 0.01. (**B**) Intra-*V. cholerae* sodium levels were measured using fluorescent dye sodium green as a measure of sodium gradient across the bacterial membrane. Monensin is a sodium-specific ionophore that affects the level of sodium chemical potential. Data shown are representative of four biological replicates, each with two technical replicates. Statistical significance compared to the absence of antibodies (No Ab) was determined by one-way analysis of variance with Dunnett’s *post hoc* test for multiple comparisons and is denoted by asterisks: ****P < 0.0001.

We have previously shown that anti-OSP antibody in liquid and semi-solid media inhibits *V. cholerae* motility, including at sub-agglutinating concentrations (22, 24). In our current analysis, we found that expression of VC2138 (*fliS*), a flagellin-specific T3SS chaperone of flagellin monomer that facilitates export and polymerization for flagellar assembly, was decreased in the presence of anti-OSP antibody and mucin (Table 1; Table S3). To directly assess *V. cholerae* motility in mucin, we used a mucin-agarose column and confirmed significant inhibition of *V. cholerae* motility in the presence of OSP-specific antibody and mucin (Fig. 5A). We next used high-speed microscopy of *V. cholerae* with fluorescently labeled flagella to specifically address whether flagellar tethering or bacterial cross-linking was occurring. We found that *V. cholerae* in the presence of anti-OSP antibody lost motility despite preserved, non-tethered flagella (Movies S1 and S2).

Since our results suggest that motility could be arrested by anti-OSP antibody even in the presence of an intact non-tethered flagellum, we explored other aspects involved in mediating *V. cholerae* motility. Motility in *V. cholerae* is dependent on the flagellar motor driven by the SMF (64), and our transcriptomic analysis identified several genes involved in the transport of sodium ions across the bacterial membrane in response to G1 in mucin, including decreased expression of VC1016 that encodes a RnfB-related protein, an ion-translocating oxidoreductase complex subunit B that is part of a membrane-bound complex that is a redox-driven ion (Na+) transporter (Table 1; Table S3). We also identified decreased expression of VCA0193 encoding a Na+/H+ antiporter in response to G1 in mucin (Table 1; Table S3). Despite the identification of such genes, we were unable to detect changes in the concentration of intracellular sodium levels in direct measurement (Fig. 5B).

### Impact of anti-OSP IgG G1 in mucin on *V. cholerae* extracellular matrix production

**Fig 6:**
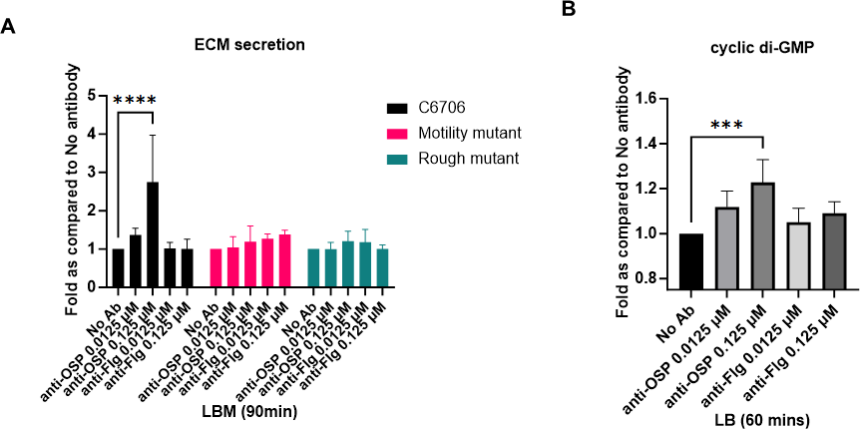
(**A**) Impact of G1 in mucin on induction of extracellular matrix by *V. cholerae*. Representative crystal violet staining assessment of *V. cholerae* C6706 wild type (black), motility mutant *V. cholerae* (deficient in stator subunit B; fuchsia), and rough mutant *V. cholerae* (deficient in perosamine synthase; teal) in the presence and absence of G1 or B12 in mucin at 37°C for 90 min. The data are representative of the results from three independent experiments, each with three replicates. Statistical significance compared to the absence of antibodies (No Ab) was determined by two-way ANOVA with Tukey’s *post hoc* test for multiple comparisons and is denoted by asterisks: ****P < 0.0001. (**B**) Impact of G1 on levels of cyclic di-GMP. Cyclic di-GMP levels were assessed via aptamer-based kit upon exposure of *V. cholerae* C6706 to G1 or B12 in LB. Data shown are expressed as mean ± standard deviation of three biological replicates. Statistical significance compared to the absence of antibodies (No Ab) was determined by one-way analysis of variance with Dunnett’s *post hoc* test for multiple comparisons and is denoted by asterisks: ***P < 0.001.

Given the strong transcriptomic evidence for the impact of G1 in mucin on *V. cholerae* biofilm formation (Table 1; Tables S3 and S4), we examined extracellular matrix (ECM) production by *V. cholerae* exposed to G1 in mucin using a crystal violet assay and *V. cholerae* C6706, as well as mutants of C6706, including a rough strain lacking OSP and a motility-deficient strain (retaining a non-functional but intact flagellum) (44, 65). G1 in mucin readily induced the expression of ECM in wild-type C6706 (Fig. 6A, black bars), which did not occur in the B12 in mucin condition. No ECM was induced in a rough *V. cholerae* mutant (not expressing OSP) in the presence of G1 in mucin (Fig. 6A, teal bars), suggesting ECM induction occurs following binding of OSP-specific antibody on *V. cholerae* OSP. In addition, ECM was also not induced in the absence of functional motility (involving a motility-deficient mutant with an intact but non-functional flagellum coated with OSP; Fig. 6A, fuchsia bars), suggesting that bacterial motility is important for OSP-specific antibody to induce ECM release by *V. cholerae*.

### Impact of anti-OSP IgG G1 in mucin on *V. cholerae* secondary messenger signaling

A well-established modulator of motility and ECM production by *V. cholerae* is the secondary messenger molecule cyclic bis-(3′-5′)-dimeric guanosine monophosphate (66–69). Since our analysis had identified an impact of G1 in mucin on *V. cholerae* motility and ECM expression, we directly assessed levels of c-di-GMP in *V. cholerae* in the presence and absence of G1 using an aptamer-based kit. Since mucin interfered with the detection of c-di-GMP (Fig. S2A), we assessed the impact of OSP-specific antibody on *V. cholerae* in media lacking mucin. We detected an increase in the levels of c-di-GMP in *V. cholerae* in the presence of LB-G1 (Fig. 6B), and this increase was not observed using the rough mutant (Fig. S2B), suggesting involvement of c-di-GMP signaling in the response of *V. cholerae* to OSP-specific antibodies.

### Impact of anti-OSP IgG G1 on *V. cholerae* colonization in human enteroids

**Fig 7:**
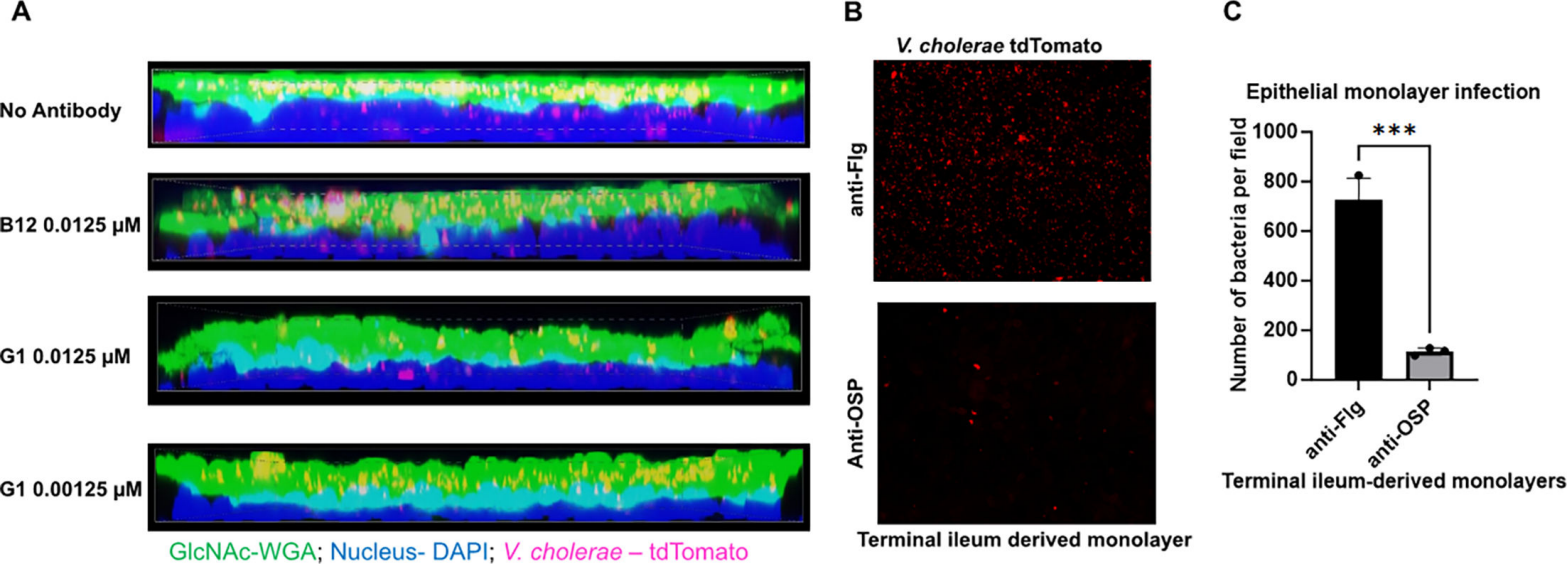
*V. cholerae* colonization and localization in a human terminal ileum-derived epithelial monolayers expressing mucus. (**A**) Representative orthogonal projection of three-dimensional rendering of Z-stacked confocal images captured (Nikon, 60×) following staining with DAPI (blue) for nuclei and lectin WGA (green) for surface and secreted glycoproteins containing N-acetyl glucosamine (GlcNAc), including in mucin, of epithelial monolayers preincubated with G1 or B12 (10 min) and overlayed with *V. cholerae* C6706 expressing tdTomato (magenta) for 30 min. Image represents a composite of the entire depth of the Z-stacked volumetric projection; this and the undulating nature of the monolayer result in a field of blue (as opposed to individual nuclei that can be discerned in the non-single-composite representation in Fig. S3), occasional bacteria “out of field” (note these rare bacteria retain their red color demonstrating they are not intracellular), and a composite teal-cyan layer reflecting both GlcNAc and nuclei between the blue (nuclei) and green (GlcNAc) fields. The main purpose of the composite is to demonstrate the impact of OSP-specific antibody on the distribution of *V. cholerae* within the mucus and surface glycoprotein layer (yellow). (**B**) *V. cholerae* colonizing the apical surface of epithelial monolayers captured via fluorescent microscopy of tdTomato-expressing bacteria in the presence of 0.0125 µM of anti-Flg B12 or anti-OSP G1. Representative apical view images of two biological replicates are shown (*N* = 2). Scale bar, 50 µM. (**C**) Quantitation of *V. cholerae* bacteria colonizing epithelial monolayers in the presence of 0.0125 µM of anti-Flg B12 (black bars) or anti-OSP G1 (gray bars) (*N* = 2). Statistical analysis was carried out using Student’s *t*-test. See Fig. S3 for more details on the enteroid model.

**Fig 8:**
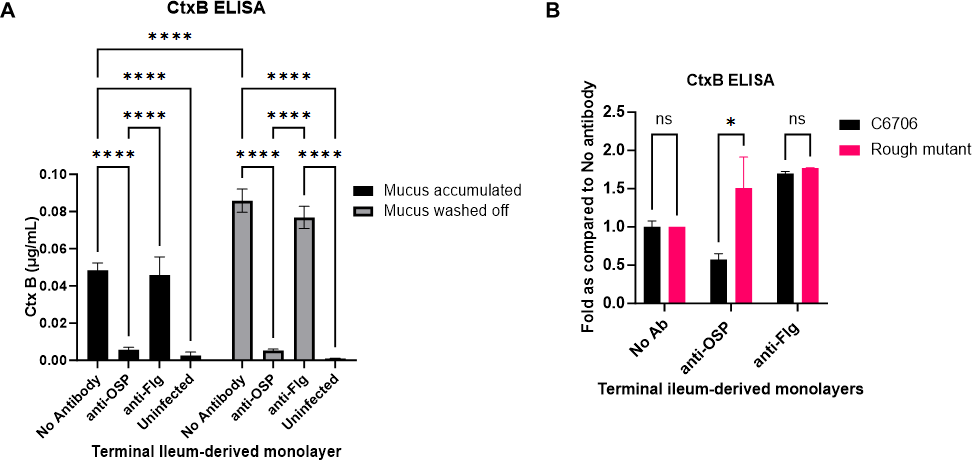
Cholera toxin in human ileum-derived epithelial monolayer upon *V. cholerae* colonization in the presence of G1 or B12. (**A**) GM1 ELISA for CT presence (via assessment of the binding subunit CtxB) in the supernatants of terminal ileum-derived monolayers infected with *V. cholerae* C6706 with (black bars) and without mucus (gray bars) and in the presence of 0.0125 µM each of G1 or B12 (*N* = 2). Statistical significance compared to the absence of antibodies (No Ab) was determined by two-way analysis of variance with Tukey’s *post hoc* test for multiple comparisons and is denoted by asterisks: ****P < 0.0001. (**B**) Assay of CT in culture supernatants of terminal ileum-derived monolayers with mucus accumulation infected with *V. cholerae* C6706 (black) or a rough mutant (lacking OSP, fuchsia) in the presence of 0.0125 µM G1 or B12 (*N* = 2). Statistical significance compared to the absence of antibodies (No Ab) was determined by two-way ANOVA with Tukey’s *post hoc* test for multiple comparisons and is denoted by asterisks: *P < 0.05.

To gain an understanding of the overall impact of the various effects of anti-OSP antibody (Fig. 1 to 6) on bacterial colonization capabilities (70), we established an epithelial monolayer colonization model using enteroids derived from human duodenal and terminal ileal adult stem cells (55). Enteroid monolayers have been used to study host interaction with enteric pathogens (55, 71, 72). Several reports have utilized enteroids to study the effects of CT and to evaluate potential inhibitors of CT (73–76). The enteroid monolayers maintain apical-to-basal polarity and barrier function and differentiate to contain multiple cell types, including goblet cells that produce mucin (a principal component of mucus) (77). We controlled the level of mucus present in our monolayers through intermittent washing. Once we established the monolayer (Fig. S3), we added antibodies G1 or B12 to the apical chamber of the Transwells (representing the luminal surface), followed by red fluorescence protein tdTomato-expressing *V. cholerae*. We quantified bacteria at the epithelial apical surface in the presence or absence of G1 or B12 following staining with 4′,6-diamidino-2-phenylindole (DAPI) for nuclei and lectin wheat germ agglutinin (WGA), which binds N-acetylglucosamine and sialic acid residues, both abundantly present in mucin glycoproteins along with several other surface glycoproteins (Fig. 7A). We quantified *V. cholerae*’s ability to colonize the epithelial surface and observed a significant reduction in the presence of OSP-specific antibody compared to anti-flagellin antibody controls (Fig. 7B and C). This was accompanied by a significant reduction in cholera toxin levels (Fig. 8A). Results were the same in sub-analyses of ileal- and duodenal-derived monolayers, and with and without accumulated mucus (Fig. 8A; Fig. S4A). No decrease in cholera toxin was noted when monolayers were exposed to a rough mutant (VC0244::Kan^r^) that does not bind G1 antibody (Fig. 8B; Fig. S4B).

## DISCUSSION

*V. cholerae* physiology is significantly altered when bacteria are exposed to anti-OSP-specific antibody in the presence of mucin. These changes result in a shift from a highly virulent and motile bacterial phenotype to a non-motile phenotype characterized by significantly decreased metabolic activity and growth, an increase in extracellular matrix associated with biofilm formation, and a decrease in the ability to colonize the human intestinal epithelial surface and express cholera toxin.

Exposure of *V. cholerae* to mucin itself alters *V. cholerae* physiology, including transcriptional downregulation of virulence-related regulons, and underscores the importance of studying the effects of anti-OSP antibody in a mucus/mucin milieu in which bacteria and antibodies would interact at the intestinal surface of an infected human. The impact of mucin on virulence gene expression has been noted previously (78–83) and may suggest that mucin acts as a signal to *V. cholerae* that it has reached its target ecological niche and should begin transcriptional alterations required to support subsequent survival and passage in diarrheal stools.

On top of these mucin-related changes, OSP-specific antibodies further altered *V. cholerae* metabolism and growth. Our data suggest that these changes were not due to agglutination, impact on bacterial viability, or impact on redox capabilities (membrane potential and ATP generation). We did note an increase in the expression of bacterial stress response genes, perhaps reflecting the impact of OSP-specific antibodies on the bacterial outer membrane. We, however, did not detect evidence for major structural damage or loss of bacterial membrane integrity in our analyses, although we did detect increased extracellular ATP from *V. cholerae* exposed to OSP-specific antibody in mucin, perhaps suggesting damage sufficient to allow leakage of small molecules. Previous analysis of a monoclonal antibody targeting the core oligosaccharide-lipid A region of *V. cholerae* LPS did suggest more substantial *V. cholerae* membrane damage than we observed (25). The different results may reflect the “deeper” lipid A membrane-associated target of the monoclonal antibody used in the previous work, compared with our OSP-specific antibody targeting the most distal component of the OSP-core-oligosaccharide-lipid A complex.

In our current analysis, we confirmed a significant impact of anti-OSP antibody on *V. cholerae* motility, including in the presence of mucin. Exposure of *V. cholerae* to mucin itself results in significant changes in transcript levels of *V. cholerae* genes involved in flagellar structure and motor components, and previous reports suggest a mechanical loss of flagellar filament and motility when *V. cholerae* penetrate the mucin layer, including at the intestinal surface (54, 84, 85). Previous analyses also suggest that after a period of a few hours within the intestinal crypts, *V. cholerae* resume their motility and undergo a “mucosal escape response,” a process that involves LuxR, LuxO, and quorum sensing (86). The “mucosal escape response” involves RpoS (a stationary phase σ factor) and HapR-dependent processes that downregulate virulence genes and upregulate motility genes, facilitating bacterial detachment (86, 87). These released bacteria are then flushed into the luminal space and exit the host within the accompanying secretory diarrheal fluid. In our current analysis, we did not detect alterations in gene expression levels for LuxO, RpoS, or HapR in the presence of OSP-specific antibody and mucin, which would not be unexpected since activity of these gene products can be regulated at post-transcriptional levels (88–90). We did detect decreased detection of the transcript of *vqmA* (VC1078, Table 1), a LuxR family transcriptional regulator involved in quorum sensing and biofilm formation (91). Our results thus suggest that OSP-specific antibodies may affect the re-acquisition of a motile phenotype after exposure to mucin, disrupting this sequence of pathophysiologic events.

We were unable to identify a definite mechanism for the inhibition of *V. cholerae* motility by OSP-specific antibodies. Because both the cell body and flagellar sheath are OSP decorated, we considered the possibility that the flagella may become tethered or cross-linked to the cell body upon exposure to anti-OSP, preventing the flagella from rotating. However, direct observation of *V. cholerae* in liquid media revealed no such events. Instead, flagellar rotation would be intermittently or permanently arrested in the presence of anti-OSP. These arrests were not observed in the absence of anti-OSP antibody. We were unable to identify a change in the sodium motive force in *V. cholerae* exposed to OSP-specific antibody in mucin. We did, however, detect an increase in the level of cyclic-di-GMP upon exposure of *V. cholerae* to anti-OSP and mucin. In *E. coli*, c-di-GMP binds YcgR, a PilZ domain protein that interacts with proton channels in the membrane to curb flagellar motor output (67, 92, 93). The YcgR-like protein of *V. cholerae* (VCA0042/PlzD) has been shown to bind c-di-GMP and could be a possible mechanism of a quick loss of motility in *V. cholerae* (94), although it was not observed to be modulated in our screen. Interestingly, high c-di-GMP also drives *V. cholerae* to transform from a curved shape that is better adapted to motility in hydrogel or high-density agar (akin to mucin) to a straight cell morphology better suited to a sessile lifestyle (95, 96). The curvature associated with motility was found to provide a competitive advantage in both infant mouse intestine and rabbit ileal models on *V. cholerae* pathogenesis (95, 96). The curved structure of *V. cholerae* is attributed to the protein encoded by gene *crvA* (VC1075), and we found decreased detection of this gene transcript in the presence of OSP-specific antibody and mucin (Table S5). This could potentially be an additional mechanism by which OSP-specific antibodies may impact *V. cholerae* motility and ability to penetrate mucin.

In *V. cholerae*, high levels of c-di-GMP also induce the formation of biofilms (97, 98). Biofilms provide protection from several environmental stresses, such as bacteriophages and host immune mediators. The conversion from planktonic to biofilm mode of *V. cholerae* involves changes in global transcriptomic profiles attributed to two transcriptional regulators: VpsR and VpsT, both of which can bind c-di-GMP (69, 99–102). We found increased expression of both gene transcripts in *V. cholerae* exposed to OSP-specific antibody in mucin. Upon binding c-di-GMP, VpsT activates transcription of target genes, including those encoding the extracellular matrix component *Vibrio* polysaccharide (*vps* gene clusters) (66, 98). We also found an increase in *vpsH* (VC0924) transcript levels in *V. cholerae* in mucin exposed to OSP-specific antibodies. Interestingly, we did not detect production/secretion of ECM in a non-motile (but smooth; containing OSP) mutant strain of *V. cholerae* exposed to OSP-specific antibodies in mucin. Similar results were also observed by Baranova and colleagues using the ZAC-3 anti-LPS antibody (25). The requirement of motility in the early stages of biofilm formation has also been previously reported by others (20, 103, 104), suggesting that induction of biofilm by *V. cholerae* must first involve cessation of active bacterial motility (105).

We did not detect a change in virulence gene expression when OSP-specific antibodies were added to mucin, apart from the significant reduction observed in *V. cholerae* exposed to mucin alone. We were, however, able to demonstrate a significant decrease in cholera toxin at the mucosal surface when *V. cholerae* were exposed to OSP-specific antibodies in the human epithelial monolayer colonization model, compared to control antibody conditions. This was accompanied by a decrease in the number of bacteria colonizing the monolayer, suggesting that a consequence of the various effects of anti-OSP on *V. cholerae* either directly (such as by inhibiting motility) or indirectly (shifting the metabolic state and lifestyle to sessile growth in an extracellular matrix) results in fewer bacteria coming into proximity to epithelial cells. The decrease in bacterial numbers, lower metabolic rate, altered physiology, and reduction in *V. cholerae* attaining proximity to the epithelial layer (70) could explain the decrease in cholera toxin that we detected in the human-derived enteroid model system.

Our study has a number of limitations. Due to availability, our *in vitro* work used porcine stomach mucin. Although this reagent is standardly used in bacterial studies as a representative mucin (106–108), we attempted to mitigate possible species-specific effects by also incorporating a human enteroid model system expressing human mucin. Our enteroid model system also does not fully recapitulate the tertiary structure or complexity of the human small intestine; however, our system does represent the first analysis of the impact of an antigen-specific antibody in a complex system containing human mucus, *V. cholerae*, and human intestinal epithelial cells. We also used a lectin to assess the mucus layer in our monolayer model; future efforts will employ reagents capable of discerning individual mucins. We chose an anti-*V*. *cholerae* flagellin antibody as a control since it is well characterized (24), was isolated from an infected human with cholera, and in light of the impact of anti-OSP antibody on *V. cholerae* motility. Since the flagellum of *V. cholerae* is sheathed and coated with OSP, future efforts could evaluate an alternative outer-membrane binding antibody. We cannot totally exclude the impact of OSP-specific antibody on bacterial agglutination in our analyses, although we used a previously characterized monoclonal OSP-specific antibody and experimental conditions to mitigate this possible effect. We also did not assess cAMP levels in our epithelial cell enteroid model to assess the impact of the decreased cholera toxin levels, although this will be a focus of future efforts. Our analysis also used a single OSP-specific IgG monoclonal antibody since it facilitated control of experimental parameters. However, at the mucosal surface where *V. cholerae* interacts with mucosal antibody, IgM (multimeric) and IgA (dimeric) antibody isotypes would be the primary isotypes present. Analysis using these antibody isotypes and across a range of affinity and other antibody attributes is a focus of ongoing work by our group, although our data to date suggest that IgM and IgA isotypes more substantially impact *V. cholerae* compared to an isogenic IgG derivative, probably reflecting the multimeric nature and perhaps altered hinge regions of these antibodies in comparison to IgG (22–24).

In summary, our findings suggest that OSP-specific antibodies have a profound effect on *V. cholerae* in complex systems containing mucin. These changes involve several key regulatory cascades, inhibition of motility, downregulation of virulence mechanisms, and physiologic shifting of bacteria to a low metabolism, amotile state within an extracellular matrix component of a biofilm. We propose that this anti-OSP antibody-mediated disruption of *V. cholerae* physiology and its associated effects on virulence explain how antibodies targeting *V. cholerae* OSP mechanistically protect against cholera in the intestinal lumen of humans in the absence of direct innate or other human immune cell bacterial engagement.

## Data Availability

The RNA sequencing data generated in this study have been deposited in the Gene Expression Omnibus (GEO) database under accession number GSE287993. The data include raw sequence reads and processed expression matrices that can be accessed at https://www.ncbi.nlm.nih.gov/geo/query/acc.cgi?acc=GSE287993.

## AFTER EPUB

*[This article was published on 12 September 2025 with an error in Jing Yan's surname. The name was corrected in the current version, posted on 17 September 2025.]*

## SUPPLEMENTAL MATERIAL

List S1 mbio.02235-25-s0001.xlsx DEGs of *V. cholerae* in various conditions.

Figure S1 mbio.02235-25-s0002.tiff Box plots of fold change in expression of target gene.

Figure S2 mbio.02235-25-s0003.tiff Cyclic di-GMP levels.

Figure S3 mbio.02235-25-s0004.tiff Schematic for generation of enteroid-derived epithelial monolayer model.

Figure S4 mbio.02235-25-s0005.tiff CT detection in supernatants of *V. cholerae* infected duodenum-derived monolayers.

Supplemental material incorporated mbio.02235-25-s0006.docx Detailed methods and supplemental tables

Legends mbio.02235-25-s0007.docx Legends for supplemental figures, tables, and movies.

Movie S1 mbio.02235-25-s0008.mp4 *V. cholerae* motility arrested by very low concentration of anti-OSP is not due to surface attachment.

Movie S2 mbio.02235-25-s0009.mp4 *V. cholerae* motility arrest at high anti-OSP concentration is not due to surface attachment.

ASM does not own the copyrights to Supplemental Material that may be linked to, or accessed through, an article. The authors have granted ASM a non-exclusive, world-wide license to publish the Supplemental Material files. Please contact the corresponding author directly for reuse.
