# Supplementary figures and images for: Human antibody targeting Vibrio cholerae O1 O-specific polysaccharide induces an amotile hypovirulent bacterial phenotype: mechanism of protection against cholera
Source: mBio. 2025 Sep 12;16(10):e02235-25. doi: 10.1128/mbio.02235-25 (PMC12505965; doi:10.1128/mbio.02235-25)

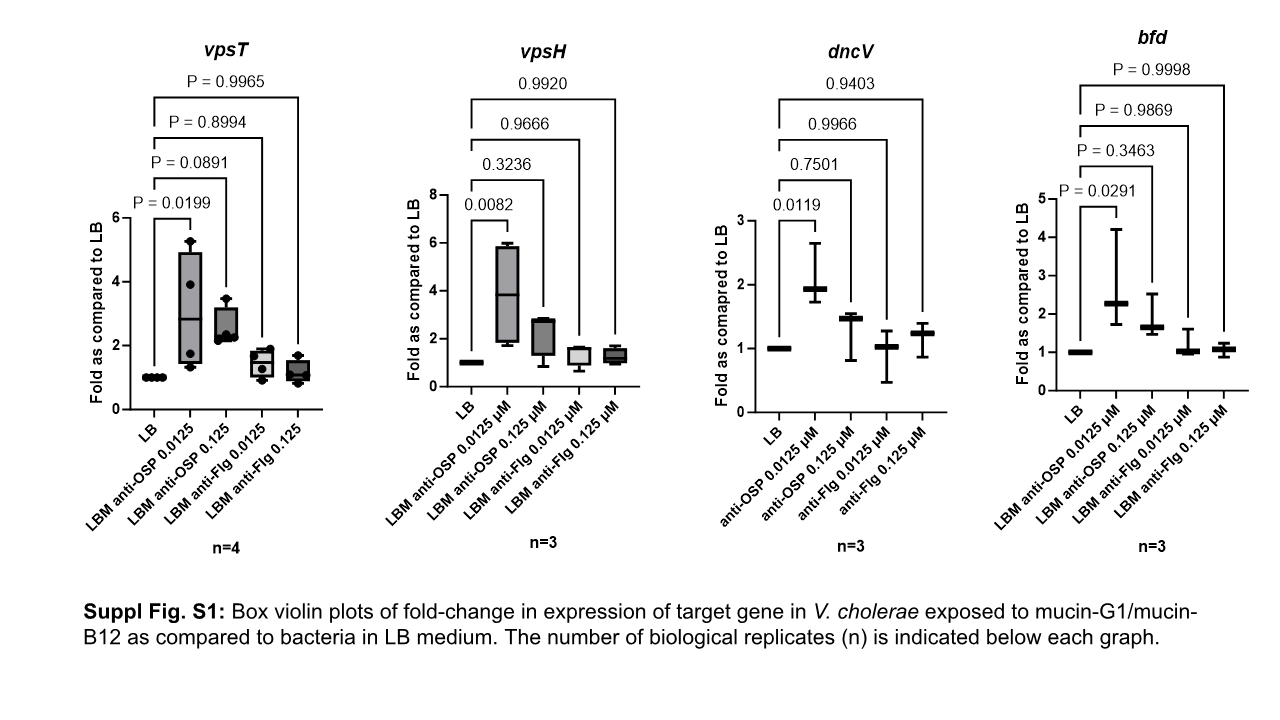

Supplement: Figure S1 — Box plots of fold change in expression of target gene. [file mbio.02235-25-s0002.tiff]

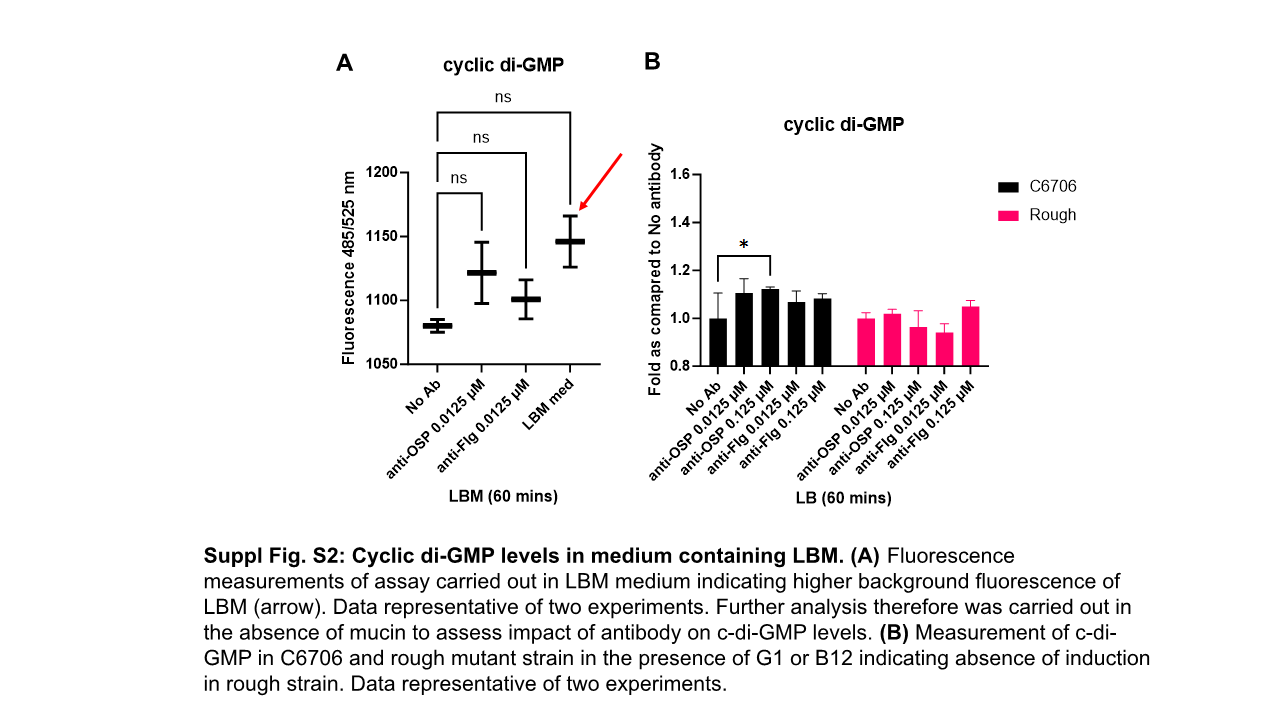

Supplement: Figure S2 — Cyclic di-GMP levels. [file mbio.02235-25-s0003.tiff]

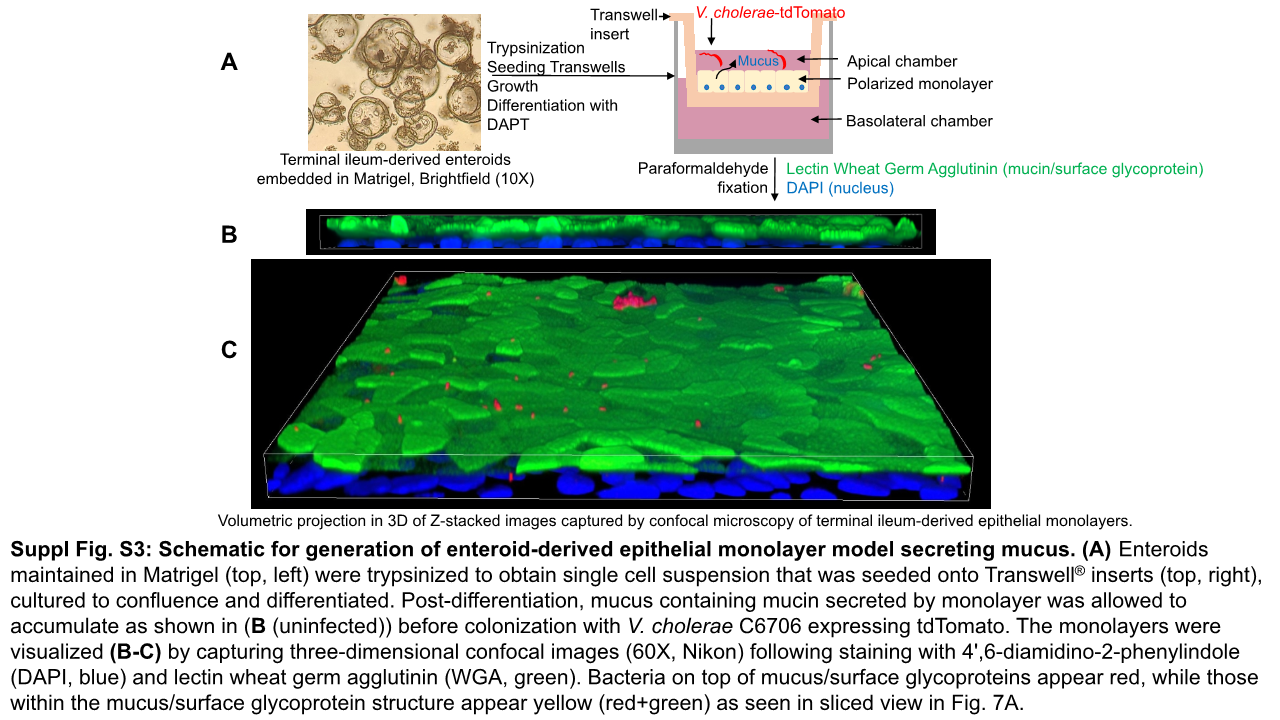

Supplement: Figure S3 — Schematic for generation of enteroid-derived epithelial monolayer model. [file mbio.02235-25-s0004.tiff]

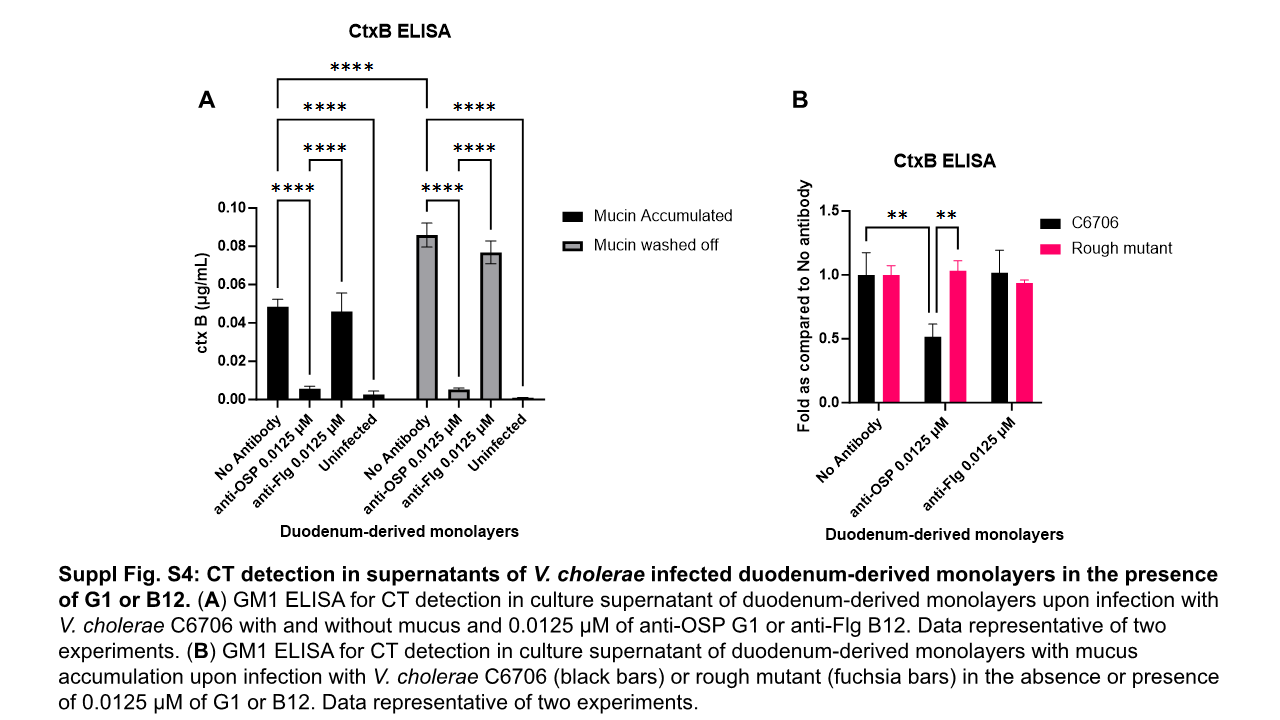

Supplement: Figure S4 — CT detection in supernatants of V. cholerae infected duodenum-derived monolayers. [file mbio.02235-25-s0005.tiff]
