# Supplementary material for: Human antibody targeting Vibrio cholerae O1 O-specific polysaccharide induces an amotile hypovirulent bacterial phenotype: mechanism of protection against cholera
Source: mBio. 2025 Sep 12;16(10):e02235-25. doi: 10.1128/mbio.02235-25 (PMC12505965; doi:10.1128/mbio.02235-25)
Supplement: Supplemental material incorporated — Detailed methods and supplemental tables [file mbio.02235-25-s0006.docx]

## **Supplemental Materials**

**Note:** Transcriptional data are available at Gene Expression Omnibus (GEO) database under accession number GSE287993 (<https://www.ncbi.nlm.nih.gov/geo/query/acc.cgi?acc=GSE287993>).

**Appendix** contains lists of all differentially expressed genes (DEGs) with a fold change of ≥ ± 1.5-fold and a false discovery rate (FDR) below 0.5 when compared to LB as control with their start and stop locations, gene product, length, logFC, P- and FDR-values denoted. Also described are the Venn Diagram analyses.

**Supplemental methods**

**Bacterial media and growth conditions:** *V. cholerae* strains (Table S1) were cultured under toxin-inducing conditions (TIC) according to conditions defined in literature (1, 2) using AKI medium (1.5% Bacto-Peptone, 0.4% yeast extract, 0.5% NaCl) supplemented with sodium bicarbonate to a final concentration of 0.3% w/v without agitation at 37 ºC for four hours. Mucin-containing medium (LBM) was prepared by adding 1% w/v porcine gastric mucin (Sigma) to LB and sterilized by autoclaving (3). M9 was obtained commercially (Sigma) while tryptone-phosphate broth (1% Bacto tryptone, 10 mM potassium phosphate, pH 7.0) (4) and tryptone broth (1% Bacto tryptone, 0.5% NaCl) were prepared in-house.

**Metabolism, growth, and viability assessment:** Metabolic activity of *V. cholerae* cultured under TIC and incubated with G1 or B12 in presence of mucin at O.D._600_ of ≤ 0.1 for 60 mins at RT was measured via MTT assay. Following antibody exposure, MTT reagent- 3-(4,5-dimethylthiazol-2-yl)-2,5-diphenyltetrazolium bromide (Molecular Probes) was added to a final concentration of 0.5 mg/mL to bacteria and incubated at RT for 30 mins. Formazan formed by bacterial metabolic activity was dissolved using 80% DMSO-20% SDS solution for 15 mins at RT. O.D. was measured at 570 nm. To assess the impact of antibodies on growth of *V. cholerae*, TIC cultures were diluted in LBM to a final O.D._600_ of ≤ 0.01 followed by addition or not of G1 or B12 antibodies in clear flat bottom 96-well plates. At 30 min intervals, O.D. was measured at 600 nm until untreated cultures reached an O.D. ~ 0.1 (non-agglutinating conditions, (5)). Viability of *V. cholerae* was assessed using a Live/Dead BacLight Bacterial Viability kit (Molecular Probes). *V. cholerae* cultured under TIC and diluted to O.D._600_ of ≤ 0.1 in LBM were treated with G1 or B12 for one hour at RT. A standard curve was generated by combining different ratios of live bacteria with bacteria killed by exposure to ethanol. Samples and standards were stained with 1:1 mix of the two dyes supplied in the kit - propidium iodide and SYTO^®^ 9 - at RT for 15 minutes in the dark. Fluorescence emissions were measured at Ex 485 nm/Em 530 nm (green) and Ex485/Em590nm (red) (BioTek Synergy 2) followed by calculating the ratio of green/red fluorescence and percentage viability.

**Assessment of bacterial membrane integrity:** Bacteria were cultured under TIC, diluted in LBM to an O.D. _600_ of ≤ 0.1 and treated with G1 or B12 antibodies at RT for 60 minutes. Following this, samples were centrifuged, and supernatants filtered through a 0.22 µm filter and processed for LPS detection using Limulus amoebocyte lysate (LAL) assay (GenScript) per manufacturer’s instructions. Bacterial supernatants were also coated onto ELISA plates (Nunc, Maxisorp) overnight at 4°C. Plates were then blocked with 1% BSA and probed with antibodies targeting i) LPS core of *V. cholerae* (432A.G1.H12, prepared in-house, diluted 1:2000), ii) membrane-bound protein zonula occludens toxin (anti-Zot antibody; Thermo Fisher, diluted 1:2000) or iii) cytoplasmic protein RNA polymerase beta subunit (anti-RNApolβ, Biolegend, diluted 1:1000) for 90 mins at 37°C. Captured antibodies were detected using goat anti-mouse (SouthernBiotech) and goat anti-rabbit (SouthernBiotech) antibodies conjugated to peroxidase (1:5000 dilution, each). Peroxidase activity was measured with substrate 2,2-azino-bis (3-ethylbenzthiazoline-6-sulfonic acid (ABTS, Sigma) and read-out was recorded kinetically using a spectrophotometer at 405 nm (SpectrMax ABS, Molecular Devices).

**Measurement of membrane electrical potential:** *V. cholerae* were cultured under TIC and resuspended in M9 Minimal Medium plus 0.5% glucose. Bacteria were loaded with fluorescent cationic dye JC-1 (5,5’,6,6’-tetrachloro-1,1’,3,3’-tetraethylbenzimidazolylcarbocyanine iodide) at a final concentration of 5 µg/mL for 15 minutes at RT. Bacteria were centrifuged and resuspended again in M9 minimal medium with glucose and allowed to recover for 15 minutes at RT. Samples were then diluted 1:10 in LBM (O.D._600_ ≤0.1) containing G1 or B12 and incubated for 30 minutes at RT. 100 µM of ionophore carbonyl cyanide m-chlorophenylhydrazine (CCCP, Abcam) was used as a control. In the presence of high membrane potential, JC-1 aggregates to form structures (J-aggregates) that fluoresce red (Ex 530nm and Em 590nm) while monomers exhibit green (Ex 485nm and Em 525 nm) fluorescence. Fluorescence measurements of monomers (Ex 485 nm and Em 525 nm) and the J-aggregates (Ex 530 nm and Em 595 nm) were measured on a fluorimeter (BioTek Synergy 2). Ratios of red/green fluorescence represent bacterial membrane potential.

**Measurement of intracellular sodium:** *V. cholerae* were cultured under TIC and loaded with Sodium Green (40 µM) in the presence of 10 mM EDTA-2K in tryptone-phosphate broth for 60 minutes at RT. Unbound dye was washed off and loaded samples were rested for 30 mins. These were diluted to O.D._600_ of ≤ 0.1 in tryptone phosphate broth containing G1 or B12 antibodies for 60 mins at RT, followed by measurement of fluorescence (Excitation 485 nm and Emission 530 nm) on a fluorimeter (BioTek Synergy 2). Monensin (Sigma), a sodium specific ionophore, was used to confirm the ability to affect the level of sodium chemical potential as a control (6, 7).

**High-speed video microscopy:** *V. cholerae* C6706 derivative strain MA042 (*fla*^AA106CS107C^ *flaB*^S106CS107C^ *flaD*^K106CS107C^ ΔVC1807::P_tac_-mScarlet-I, Spec^R^, ΔcheY3) was inoculated from frozen stock into lysogeny broth (LB) and cultured overnight at 30°C with shaking. Overnight culture was regrown in M9 minimal media (Sigma) supplemented with 2 mM MgSO4 (JT Baker), 100 µM CaCl2 (JT Baker), and 0.5% glucose, cultured at 37°C with shaking for 3.5 hours, until OD600 reached 0.8. To fluorescently label flagella, culture was centrifuged at 4000 rcf for 4 minutes, and the supernatant was removed and replaced with Alexa Fluor 488 C5-maleimide (ThermoFisher) at a concentration of 25 µg/mL (diluted in tryptone broth: 1% Bacto tryptone, 0.5% NaCl). Cells were resuspended by gentle pipetting. This suspension was incubated at room temperature for 10 minutes before being washed three times by centrifuging cells as before, replacing supernatant with fresh tryptone broth, and gently pipette mixing the cells to resuspend. All cell transfers were accomplished with pipette tips that had been pre-cut to widen the opening and prevent flagellar damage due to shear.

Immediately before imaging, cells were mixed in a 1:1 ratio with anti-OSP antibody to achieve the concentration given in the figure legend. Cells were transferred into 10 micron-tall PDMS microchannels with glass bottoms for imaging using EPI-illumination on a Nikon Ti2-E. Simultaneous dual color video was achieved with a Cairn OptoSplit II placed at the end of the light path just before the EMCCD, an Andor Ixon life 888.

**Measurement of ATP:** *V. cholerae* bacteria were cultured under TIC, diluted in LBM with or without G1 or B12 antibodies to a final O.D._600_ of ≤ 0.1, and incubated at RT for 60 minutes. To measure total (intracellular and extracellular) ATP, following incubation with G1 or B12, BacTiter-Glo^TM^ Reagent was added to bacterial suspension and luminescence was measured after a 5 min incubation at RT (BioTek Synergy 2). The ionophore CCCP (100 µM) was used as a control. Extracellular ATP (eATP) released into culture medium was assayed from centrifuged supernatants obtained after treatment of bacteria. Supernatants were passed through a 0.22 µm filter and mixed with BacTiter-Glo^TM^ Reagent as described above.

**Crystal violet assay:** *V. cholerae* C6706 wild-type, motility mutant, and rough strain (Table S1) were cultured under TIC and diluted to a final O.D._600_ of ≤ 0.01 in LBM. These were then incubated with or without G1 or B12 antibodies in polystyrene 96 well plates (Costar) for 90 mins at 37 ºC without agitation. Cultures were aspirated off and plates were washed with PBS, fixed with methanol for 15 minutes and air dried. Once dried, plates were stained with 0.01% v/v crystal violet (CV) for 5 mins, rinsed with water and allowed to air-dry overnight. CV staining was solubilized in ethanol (30 minutes) and measured using a spectrophotometer (SpectraMax ABS, Molecular Devices) at 570 nm.

**Cyclic di-GMP measurement:** *V. cholerae* were cultured under TIC and diluted in 1 mL LB with or without G1 or B12 antibodies such that the final O.D._600_ was ≤ 0.1, followed by incubation at RT for 60 mins. Bacteria were centrifuged and resuspended in 100 µL LB of which 5 µL was added to assay mix prepared from components supplied with the kit (Lucerna Technologies), while the remaining sample was used to measure O.D._600_ for normalization. The assay mixtures with treated bacteria were transferred to black assay plates (Costar) and incubated in the dark for 30 minutes after which fluorescence was measured at Ex 485 nm and Em 525 nm (BioTek Synergy 2). According to manufacturers, the kit is highly selective for c-di-GMP with no interference from common counter ligands, detects as low as 50nM of c-di-GMP and has a broad dynamic range.

**Human enteroid-derived polarized epithelial monolayers:** Enteroids established from human adult intestinal stem cells from the terminal ileum and duodenum were maintained in Matrigel (Corning) in a 1:1 mixture of L-WRN (Wnt3a, R-spondin-3 and Noggin) conditioned medium (prepared in-house) and Intestinal Stem Cell Media containing A83-01 (Sigma) and Y-27632 (Calbiochem), as described previously (8–10). To generate monolayers, enteroids were trypsinized to obtain single cell suspensions, and 100,000 cells were then seeded on 6.5 mm Transwells® inserts with 0.4 µm pore size (Corning) in 1:1 medium supplemented with Y-27632 and incubated at 37 °C with 5% CO_2_. Culture medium was replaced every two days and generation of the monolayers was monitored by measuring the trans-epithelial electrical resistance (TEER, EVOM2, World Precision Instruments) and via microscopic observation (Zeiss). Monolayers were differentiated using γ-secretase inhibitor DAPT (N-[N-(3, 5-difluorophenacetyl)-l-alanyl]-s-phenylglycinet-butyl ester, Calbiochem) with Wnt gradient for 48 hours, as previously described (8). After differentiation, mucus secreted by monolayers was allowed to accumulate for 3-5 days and confirmed using three-dimensional confocal microscopy (Nikon) of paraformaldehyde-fixed monolayers as described below and represented in Suppl Fig. S3.

**Immunofluorescence:** Intestinal monolayers in Transwells® following infection were fixed by incubation with 4% paraformaldehyde overnight at 4 °C. After washing the fixative off, monolayers were blocked with 5% BSA for one hour at RT. The mucus layer containing mucin produced by the monolayers was visualized by incubation with fluorescein conjugated lectin Wheat Germ Agglutinin (WGA, Vector Laboratories) for one hour at RT as described by Nystrom and colleagues (11). WGA exhibits a high affinity for specific sugar residues that are commonly present in sialic acid (N-acetylneuraminic acid) and N-acetyl glucosamine (GlcNAc), commonly found in mucins and surface glycoproteins. The cells were counterstained with nuclear stain DAPI (Sigma) for 15 mins before being mounted on a glass slide with FluorSave Reagent (EMD Millipore). Z-stack images were captured via confocal microscopy (Nikon Eclipse Ti Inverted Confocal microscope) and 2D slices captured at different depths merged to obtain volumetric projection in 3D (Fig. S3C). Orthogonal projections of the 3D rendering showcasing data across the entire depth of the image and offering a comprehensive visualization of the 3D structure from all angles are depicted in Fig. 7A.

For quantitation of infection (Fig. 7B-C), single plane images of monolayers infected with tdTomato expressing *V. cholerae* were captured using a fluorescence microscope (Nikon).

**GM1 ELISA for Cholera Toxin**: ELISA plates (Nunc, Maxisorp) were coated with monosialoganglioside GM_1_ (Sigma, 1 µg/mL) in 50 mM carbonate-bicarbonate buffer (15 mM disodium carbonate, 35 mM sodium bicarbonate) overnight at RT. The following day wells were blocked with 1% bovine serum albumin (BSA, Sigma) prepared in PBS for 1 hr at 37 °C. Samples and cholera toxin beta subunit (CtxB, Sigma) standards were loaded onto plates and incubated for 90 mins at 37°C or overnight at 4 °C in a humidified chamber. Bound CT was detected by incubation with rabbit anti-CtxB antibody (Sigma, diluted 1:1000) for 90 mins at 37°C. Captured antibody was tagged with goat anti-rabbit antibody conjugated to HRP (Sigma, diluted 1:5000) for 90 mins at 37°C. Peroxidase activity was developed with substrate ABTS and measured kinetically at 405 nm (SpectraMax, Molecular Devices).

**Quantitative real-time PCR analysis**: Assessment of changes in expression of virulence genes and validation of key targets identified by RNA sequencing was carried out by q-PCR for selected targets. One µg of RNA from *V. cholerae* C6706 exposed to LB or LBM-G1/LBM-B12 was treated with DNase (Promega) and converted to c-DNA using iScript c-DNA synthesis kit (Bio-rad) per manufacturer’s instructions. The q-PCR assays were performed using the CFX Connect Real-Time System (Bio-Rad) and PerfeCTa SYBR Supermix (Andwin Scientific). The PCR cycle was 95°C for 10 min and 40 cycles of 95°C for 15 sec, 60°C for 30 sec, and 72°C for 30 sec, followed by melt curve analysis at 65°C for 05 sec and 95°C for 15 sec. The *rpoD* or 16s gene was used as an endogenous reference gene, and the relative changes in gene expression were calculated based on the delta Ct method. The primer sequences used are shown in Table S6 below.

**Supplemental Tables**

**Table S1:** **Bacterial strains used in the study.**

| **Strain** | **Genotype/description** | **Phenotype** | **Source** |
| --- | --- | --- | --- |
| *V. cholerae* C6706 | *V. cholerae* O1 El Tor strain C6706; Inaba; lacZ-; Str resistant | Wild type | Originally in 1991 in Peru. (12, 13) |
| *V. cholerae* C6707 tdTomato | *V. cholerae* O1 El Tor strain C6706; Inaba; lacZ::tdTomato; Str^r^ | *V. cholerae* constitutively expressing codon-optimized version of tdTomato gene under the control of lac promoter. | (14) |
| *V. cholerae* VC0244::Kan^r^ | *V. cholerae* O1 El Tor strain C6706; Inaba; lacZ-; Str-resistant; Kan-resistant | Rough strain; does not produce O-specific polysaccharide of LPS due to absence of functional perosamine synthase; motile | (15) |
| *V. cholerae* pomB::Kan^r^ | *V. cholerae* O1 El Tor strain C6706; Inaba; lacZ-; Str-resistant; Kan-resistant | Motility mutant with intact flagella; Mutation in the B subunit of stator complex; intact OSP synthesis | (16) |
| *V. cholerae* MA042 | *V. cholerae* (*flaA*^A106CS107C^ *flaB*^S106CS107C^ *flaD*^K106CS107C^ ΔVC1807::P_tac_-mScarlet-I, Spec-resistant, ΔcheY3) | *V. cholerae* expressing red fluorescent protein mScarlet-I and containing cysteine substitutions in flagellin genes *flaA*, *flaB* and *flaD* that allow flagellum labeling using fluorescent maleimide dyes. | This study – Jung-Shen Benny Tai and Jing Yan |

Str: Streptomycin

Kan: Kanamycin

Spec: Spectinomycin

lacZ: β-galactosidase

**Table S2: Partial list of genes whose transcript amount was altered in presence of mucin (LB vs LBM) from two independent biological replicates.**

| **Gene** | **Annotation** | **Description** | **logFC** | **Fold change** |
| --- | --- | --- | --- | --- |
| **Virulence** | |  |  |  |
| VC0984 | *toxR* | cholera toxin transcriptional activator | -1.648 | 0.319 |
| VC0827 | *tcpH* | toxin co-regulated pilus biosynthesis protein H | -1.616 | 0.326 |
| VC1456 | *ctxB* | cholera enterotoxin subunit B | -0.932 | 0.524 |
| VC0831 | *tcpC* | toxin co-regulated pilus biosynthesis protein C | -0.773 | 0.585 |
| VC0829 | *tcpB* | toxin co-regulated pilus biosynthesis protein B | -0.895 | 0.538 |
| VC0835 | *tcpT* | toxin co-regulated pilus biosynthesis protein T | -0.978 | 0.508 |
| VC0833 | *tcpD* | toxin co-regulated pilus biosynthesis protein D | -0.990 | 0.503 |
| VC0834 | *tcpS* | toxin co-regulated pilus biosynthesis protein S | -0.999 | 0.500 |
| VC0825 | *tcpI* | toxin co-regulated pilus biosynthesis protein I | -1.356 | 0.391 |
| VC0832 | *tcpR* | toxin co-regulated pilus biosynthesis protein R | -2.064 | 0.239 |
| VC1450 |  | RTX toxin activating protein | -1.784 | 0.290 |
| VC1448 | *rtxB* | RTX toxin transporter | -3.069 | 0.119 |
| VC1446 | *rtxE* | toxin secretion transporter | -1.036 | 0.488 |
| VC1447 |  | RTX toxin transporter | -1.461 | 0.363 |
| VC0840 | *acfB* | accessory colonization factor AcfB | -0.854 | 0.553 |
| VC0844 | *acfA* | accessory colonization factor AcfA | -1.393 | 0.381 |
|  |  |  |  |  |
| **Flagellar biosynthesis** | | |  |  |
| VC2188 | *flaA* | flagellin | -0.677 | 0.625 |
| VC2143 | *flaD* | flagellin | -0.878 | 0.544 |
| VC2187 | *flaC* | flagellin | 0.885 | 1.846 |
| VC2203 | *flgA* | flagellar basal body P-ring biosynthesis protein FlgA | 1.260 | 2.395 |
| VC2601 | *motX* | sodium-type flagellar protein MotX | 1.171 | 2.252 |
| VC2141 | *flaG* | flagellar protein FlaG | 0.945 | 1.925 |
| VC2125 | *fliN* | flagellar motor switch protein | 0.874 | 1.833 |
| VC2127 | *fliL* | flagellar basal body protein FliL | 0.824 | 1.770 |
| VC0097 | *fliL* | flagellar basal body protein FliL | -0.766 | 0.588 |
| VC2122 | *fliQ* | flagellar biosynthesis protein FliQ | 0.718 | 1.645 |
| VC2194 | *flgH* | flagellar basal body L-ring protein | 0.677 | 1.599 |
| VC2069 | *flhA* | flagellar biosynthesis protein FlhA | 0.658 | 1.578 |
| VC2139 | *flaI* | flagellar rod protein FlaI | 0.648 | 1.568 |
| VC2200 | *flgB* | flagellar basal-body rod protein FlgB | 0.637 | 1.555 |
| VC0892 | *pomA* | flagellar motor protein PomA | 0.933 | 1.910 |
|  |  |  |  |  |
| **Chemotaxis** | |  |  |  |
| VC1602 | cheV | chemotaxis protein CheV | 1.418 | 2.673 |
| VC1316 | cheY | chemotaxis protein CheY | 2.805 | 6.988 |
| VC2006 | *cheV* | chemotaxis protein CheV | 1.172 | 2.254 |
| VCA0189 | *CheC* | response regulator | 0.819 | 1.765 |
| VC2062 | cheB | chemotaxis-specific methylesterase | 0.667 | 1.588 |
|  |  |  |  |  |
| VCA1069 |  | methyl-accepting chemotaxis protein | 3.179 | 9.058 |
| VC1898 |  | methyl-accepting chemotaxis protein | 2.757 | 6.762 |
| VCA0773 |  | methyl-accepting chemotaxis protein | 2.583 | 5.990 |
| VC0282 |  | methyl-accepting chemotaxis protein | 2.175 | 4.517 |
| VC0449 |  | methyl-accepting chemotaxis protein | 1.694 | 3.236 |
| VC2439 |  | methyl-accepting chemotaxis protein | 1.339 | 2.530 |
| VCA0176 |  | methyl-accepting chemotaxis protein | 1.300 | 2.462 |
| VC2161 |  | methyl-accepting chemotaxis protein | 0.889 | 1.852 |
| VC1313 |  | methyl-accepting chemotaxis protein | -0.981 | 0.507 |
| VCA0974 |  | methyl-accepting chemotaxis protein | -1.335 | 0.396 |
| VCA0864 |  | methyl-accepting chemotaxis protein | -1.717 | 0.304 |
| VC1413 |  | methyl-accepting chemotaxis protein | -2.301 | 0.203 |
|  |  |  |  |  |
| **Twitching motility** | |  |  |  |
| VC1612 | pilF | fimbrial biogenesis and twitching motility protein | 4.238 | 18.864 |
| VC0463 |  | twitching motility protein PilT | 1.240 | 2.362 |
| VC0462 |  | twitching motility protein PilT | 0.959 | 1.944 |
|  |  |  |  |  |
| **MSHA pili** | |  |  |  |
| VC0407 |  | MSHA biogenesis protein MshF | 1.202 | 2.301 |
| VC0401 |  | MSHA biogenesis protein MshK | 0.741 | 1.671 |
| VC0408 |  | MSHA pilin protein MshB | 0.607 | 1.523 |
|  |  |  |  |  |
| **Sialic acid metabolism** | | |  |  |
| VC1777 | *Sia P* | hypothetical protein | 2.904 | 7.483 |
| VC1778 | *SiaQ* | hypothetical protein | 2.883 | 7.378 |
| VC1784 | nanH | neuraminidase/ sialidase | 1.703 | 3.257 |
| VC0994 | nagA | N-acetylglucosamine-6-phosphate deacetylase | 0.853 | 1.806 |
| VC1532 | nagK | N-acetyl-D-glucosamine kinase | 2.708 | 6.534 |
|  |  |  |  |  |
| **PTS system** | |  |  |  |
| VCA0516 | fruA | PTS system fructose-specific transporter subunit IIBC | 3.572 | 11.892 |
| VCA0518 | fruB | PTS system fructose-specific transporter subunit IIA/HPr protein | 3.870 | 14.619 |
| VC1821 | frwBC | PTS system fructose-specific transporter subunit IIBC | 3.664 | 12.673 |
| VCA0517 |  | 1-phosphofructokinase | 3.716 | 13.144 |
| VC1826 | mapP | PTS system fructose-specific transporter subunit IIABC | 3.456 | 10.975 |
| VC1822 |  | PTS system fructose-specific transporter subunit IIABC | 2.792 | 6.926 |
| VC0207 | murP | PTS system N-acetylmuramic acid transporter subunits IIBC | 2.790 | 6.918 |
| VCA0653 |  | PTS system sucrose-specific transporter subunit IIBC | 2.709 | 6.540 |
| VC0910 |  | PTS system trehalose(maltose)-specific transporter subunits IIBC | 2.487 | 5.605 |
| VC2013 |  | PTS system glucose-specific transporter subunits IIBC | 2.106 | 4.305 |
| VC1820 |  | PTS system fructose-specific transporter subunit IIA | 1.939 | 3.834 |
| VC1823 |  | PTS system fructose-specific transporter subunit IIB | 1.859 | 3.629 |
| VC0995 |  | PTS system N-acetylglucosamine-specific transporter subunit IIABC | 1.696 | 3.240 |
| VC1824 |  | PTS system nitrogen regulatory subunit IIA | 1.573 | 2.975 |
| VCA1045 |  | PTS system mannitol-specific transporter subunit IIABC | 1.443 | 2.718 |
| VC0672 |  | fused phosphoenolpyruvate-protein phosphotransferase PtsP/GAF domain-containing protein | 0.672 | 1.593 |
| VC2531 |  | PTS system nitrogen regulatory subunit IIA | -0.704 | 0.614 |
|  |  |  |  |  |
| **Citrate metabolism** | |  |  |  |
| VC0791 | - | sensor kinase citA | 3.191 | 9.134 |
| VC0790 | - | transcriptional regulator CitB | 2.220 | 4.657 |
| VC0794 | *oadG* | hypothetical protein [ operon citS-oadGAB-citAB] | 8.442 | 347.726 |
| VC0795 | *citS* | citrate/sodium symporter [operon citCDEFG] | 8.235 | 301.228 |
| VC0796 | *citC* | citrate (pro-3S)-lyase ligase [operon citCDEFG] | 10.816 | 1803.129 |
| VC0798 | *citE* | citrate lyase subunit beta [operon citCDEFG] | 4.872 | 29.287 |
| VC0799 | *citF* | citrate lyase subunit alpha [operon citCDEFG] | 4.954 | 31.001 |
| VC0800 | *citX* | apo-citrate lyase phosphoribosyl-dephospho-CoA transferase | 3.823 | 14.153 |
| VC0801 | *citG* | 2-(5''-triphosphoribosyl)-3'-dephosphocoenzyme-A synthase [operon citCDEFG] | 6.351 | 81.628 |

**Table S3: List of *V. cholerae* C6706 genes whose transcript amount was altered by both concentrations of G1 in LBM compared with LB alone from two independent biological replicates. Refer to Results section and Appendix 1**

|  |  |  | **mucin-G1 0.0125 µM** | | **mucin-G1 0.125 µM** | |
| --- | --- | --- | --- | --- | --- | --- |
| **Gene** | **Annotation** | **Description** | **logFC** | **Fold change** | **logFC** | **Fold change** |
|  |  |  |  |  |  |  |
| **Metabolism** |  |  |  |  |  |  |
| VC1507 |  | phospho-2-dehydro-3-deoxyheptonate aldolase/ Chorismate biosynthesis | -0.61 | 0.66 | -0.70 | 0.61 |
| VC0215 | *coaBC* | bifunctional phosphopantothenoylcysteine decarboxylase/phosphopantothenate synthase/ Coenzyme A biosynthesis | -0.63 | 0.64 | -0.67 | 0.63 |
| VC1167 | *tdk* | thymidine kinase/ Salvage pathway of DNA synthesis | -0.75 | 0.59 | -0.67 | 0.63 |
| VC1061 |  | cysteine synthase | -0.83 | 0.56 | -0.64 | 0.64 |
| VC2152 | *dapE* | succinyl-diaminopimelate desuccinylase | -0.66 | 0.63 | -0.70 | 0.62 |
| VC2256 | *upps* | undecaprenyl diphosphate synthase/ Biogenesis of cell wall | -0.59 | 0.66 | -0.63 | 0.65 |
| VCA0898 | *gnd* | 6-phosphogluconate dehydrogenase/ Pentose phosphate pathway | 0.62 | 1.54 | 0.66 | 1.58 |
| VC0364 | *bfd* | bacterioferritin-associated ferredoxin/ Iron storage | 0.68 | 1.60 | 1.01 | 2.01 |
| VCA0192 | *ldh* | D-lactate dehydrogenase | 0.73 | 1.66 | 0.72 | 1.65 |
| VC1440 | *CcoQ* | cytochrome c oxidase subunit CcoQ/ Microaerobic respiration | 0.78 | 1.72 | 0.61 | 1.52 |
| VC1047 | *fadJ* | multifunctional fatty acid oxidation complex subunit α | 0.97 | 1.96 | 0.90 | 1.87 |
| VC2240 | *padC* | decarboxylase | 1.61 | 3.06 | 1.22 | 2.33 |
| VCA0875 | *dsdA* | D-serine dehydratase | 1.70 | 3.26 | 1.62 | 3.08 |
|  |  |  |  |  |  |  |
| **Transport** |  |  |  |  |  |  |
| VC0191 |  | putative throenine trasnporter | -1.26 | 0.42 | -0.99 | 0.50 |
| VC0992 | *kefB* | glutathione-regulated potassium-efflux system protein KefB | -0.98 | 0.51 | -1.07 | 0.48 |
| VC1658 |  | serine transporter | -0.95 | 0.52 | -1.02 | 0.49 |
| VC2381 |  | vit B12 trasnport system | -0.88 | 0.54 | -1.10 | 0.47 |
| VC1524 |  | ABC transporter permease | -0.84 | 0.56 | -0.99 | 0.50 |
| VC2555 | *zrgE* | Putative zinc related gene | -0.76 | 0.59 | -0.70 | 0.61 |
| VC1525 |  | ABC transporter ATP-binding protein | -0.75 | 0.60 | -0.78 | 0.58 |
| VCA0193 |  | Na+/H+ antiporter | -0.67 | 0.63 | -0.61 | 0.66 |
| VC1016 | *rnfB* | Ion-translocating oxidoreductase complex subunit B | -0.64 | 0.64 | -0.67 | 0.63 |
| VC1695 |  | formate transporter 1 | -0.60 | 0.66 | -0.63 | 0.65 |
| VC1546 | *exb2* | TonB system transport protein ExbB2 | 0.60 | 1.51 | 0.65 | 1.57 |
| VC1279 | *opuD* | BCCT family transporter | 0.63 | 1.55 | 0.73 | 1.66 |
| VC2724 | *epsM* | cholera toxin secretion protein EpsM | 0.67 | 1.59 | 0.87 | 1.82 |
|  |  |  |  |  |  |  |
| **Flagellar Assembly** | |  |  |  |  |  |
| VC2138 | *fliS* | chaperone protein fliS | -0.82 | 0.57 | -0.66 | 0.63 |
|  |  |  |  |  |  |  |
| **Biofilm formation** | |  |  |  |  |  |
| VCA0952 | *vpsT* | LuxR family transcriptional regulator | 0.87 | 1.82 | 0.84 | 1.78 |
| VC0924 | *vpsH* | capsular polysaccharide biosynthesis protein CapK | 0.71 | 1.63 | 0.94 | 1.92 |
| VCA1078 | *vqmA* | LuxR family transcriptional regulator | -0.60 | 0.66 | -0.63 | 0.65 |
|  |  |  |  |  |  |  |
| **Bacterial defense** | |  |  |  |  |  |
| VC0179 | dnvC | Dinucleotide cyclase in *Vibrio* | 0.59 | 1.50 | 0.65 | 1.56 |
| VC0181 |  | CBASS | 0.61 | 1.52 | 0.60 | 1.51 |
| VCA0019 | vasW | Type 6 secretion system | 1.77 | 3.42 | 1.55 | 2.92 |
| VCA0681 |  | 3'3-cGAMP phosphodiesterase | 1.94 | 3.85 | 1.84 | 3.59 |
|  |  |  |  |  |  |  |
| **Transcriptional regulation** | |  |  |  |  |  |
| VC0486 |  | DeoR family transcriptional regulator | -0.81 | 0.57 | -0.96 | 0.51 |
| VC1118 |  | Putative transcriptional regulator | 1.31 | 2.48 | 1.24 | 2.37 |
| VCA0982 |  | LysR family transcriptional regulator | 1.50 | 2.83 | 1.78 | 3.43 |
|  |  |  |  |  |  |  |
| **Protein synthesis modification** | | |  |  |  |  |
| VC1388 | lplA | lipoate-protein ligase A | 0.62 | 1.53 | 0.77 | 1.70 |
| VC2409 | rsmH | S-adenosyl-methyltransferase MraW | 0.64 | 1.56 | 0.59 | 1.50 |
| VC2774 | rsmG | 16S rRNA methyltransferase GidB | 0.66 | 1.58 | 0.59 | 1.50 |
|  |  |  |  |  |  |  |
| **Stress response** |  |  |  |  |  |  |
| VC0445 | surA | survival protein SurA | -0.59 | 0.66 | -0.59 | 0.66 |
| VC1676 | pspC | phage shock protein C | 1.15 | 2.22 | 1.18 | 2.26 |
|  |  |  |  |  |  |  |
| **Hypothetical protein** | |  |  |  |  |  |
| VC1941 |  |  | -0.66 | 0.63 | -0.65 | 0.64 |
| VC0559 |  |  | -0.63 | 0.65 | -0.73 | 0.60 |
| VC0519 |  |  | -0.61 | 0.66 | -0.66 | 0.63 |
| VCA0424 |  |  | 0.59 | 1.51 | 0.71 | 1.64 |
| VC2455 |  |  | 0.67 | 1.59 | 0.72 | 1.65 |
| VC0220 |  |  | 0.72 | 1.64 | 0.67 | 1.59 |
| VCA0125 |  |  | 0.76 | 1.69 | 1.30 | 2.47 |
| VCA0409 |  |  | 1.13 | 2.19 | 1.17 | 2.25 |
| VCA0410 |  |  | 1.34 | 2.53 | 1.39 | 2.62 |
| VCA0464 |  |  | 1.72 | 3.31 | 2.02 | 4.05 |
|  |  |  |  |  |  |  |

**Table S4: List of genes whose transcript amount was altered exclusively in mucin-G1 0.0125 µM compared with LB alone from two independent biological replicates. Refer to Results section and Appendix 1**

| **Gene** | **Annotation** |  | **Description** | **logFC** | **FOLD** | **Associated pathways** |
| --- | --- | --- | --- | --- | --- | --- |
|  |  |  |  |  |  |  |
| VCA0645 |  |  | hypothetical protein | 2.57 | 5.92 | Hypothetical protein |
| VCA0685 |  |  | iron(III) ABC transporter substrate-binding protein | 2.33 | 5.03 | Transport |
| VC1031 |  |  | inosine monophosphate dehydrogenase-like protein | 1.11 | 2.16 | Inosine monophosphate dehydrogenase-related protein |
| VC2641 | *argH* |  | argininosuccinate lyase | 1.00 | 2.00 | Arginine biosynthesis |
| VCA0505 |  |  | acetyltransferase | 0.93 | 1.91 | Acetyltransferase, putative |
| VC0106 |  |  | hypothetical protein | 0.89 | 1.85 | hypothetical protein |
| VC2749 |  |  | nitrogen regulation protein NR(I) | 0.88 | 1.84 | Signal transduction/ Biofilm |
| VC0482 |  |  | chromosome replication initiation inhibitor protein | 0.86 | 1.82 | Regulation of trasncription/DNA interaction |
| VC2384 |  |  | hypothetical protein | 0.79 | 1.73 | Hypothetical protein |
| VC1675 |  |  | multidrug resistance protein | 0.73 | 1.66 | Trasnport/efflux/secretion |
| VC2456 |  |  | hypothetical protein | 0.68 | 1.60 | Hypothetical protein |
| VC0665 | *vpsR* |  | Fis family transcriptional regulator | 0.66 | 1.58 | cyclic-di-GMP-binding transcriptional regulator VpsR |
| VC0470 | *dns* |  | extracellular deoxyribonuclease | 0.66 | 1.58 | Endonuclease/BIOFILM formation |
| VC2343 |  |  | DNA repair protein RadA | 0.63 | 1.55 | DNA Metabolism/DNA repair |
| VC2272 |  |  | transcriptional regulator NrdR | 0.62 | 1.54 | Transcriptional reguation |
| VC2157 | *dapA* |  | dihydrodipicolinate synthase | 0.62 | 1.53 | Amino acid biosynthesis |
| VC0350 |  |  | HflC protein | 0.62 | 1.53 | Intergral to membrane/ Protein interactions |
| VC2116 | *aroC* |  | chorismate synthase | 0.61 | 1.53 | Aromatic amino acid biosynthesis |
| VCA0392 | *higA* |  | antidote protein | 0.61 | 1.53 | Sequence-specific DNA binding |
| VC0648 | *NlpI* |  | lipoprotein NlpI | 0.59 | 1.50 | Signal peptidase II activity |
| VC0116 | *HemN* |  | coproporphyrinogen III oxidase | -0.59 | 0.67 | Biosynthesis of cofactors, prosthetic groups and carriers Porphysin biosynthesis |
| VC1299 |  |  | 6-pyruvoyl tetrahydrobiopterin synthase | -0.61 | 0.66 | Protein synthesis tRNA and rRNA base modification |
| VCA0274a | *Cah* |  | hypothetical protein | -0.62 | 0.65 | Hypothetical protein |
| VCA1021 |  |  | hypothetical protein | -0.63 | 0.65 | Hypothetical protein |
| VC0565 | *degS* |  | protease DegS | -0.63 | 0.64 | Periplasmic serine peptidase/Stress response |
| VC0766 |  |  | exodeoxyribonuclease VII large subunit | -0.64 | 0.64 | DNA degradation |
| VC0306 | *trxA* |  | thioredoxin | -0.65 | 0.64 | Energy metabolism/Electron trasnport |
| VC1847 | *ruvC* |  | Holliday junction resolvase | -0.66 | 0.63 | DNA metabolism DNA replication, recombination and repair |
| VC0123 | *cyaY* |  | frataxin-like protein | -0.73 | 0.60 | Biosynthesis of co-factors, prsthetic groups and carriers |
| VC0008 |  |  | amino acid ABC transporter ATP-binding protein | -0.85 | 0.56 | Amino acid trasnport |
| VC0942 |  |  | hypothetical protein | -0.89 | 0.54 | hypothetical protein |
| VC2002 |  |  | hypothetical protein | -0.95 | 0.52 | hypothetical protein |
| VCA0076 |  |  | hypothetical protein | -1.06 | 0.48 | hypothetical protein |

**Table S5: List of genes whose transcript amount was altered exclusively in mucin-G1 0.125 µM compared to LB alone from two independent biological replicates. Refer to Results section and Appendix 1**

| **Gene** | **Annotation** | **Description** | **logFC** | **FOLD** | **Associated pathways** |
| --- | --- | --- | --- | --- | --- |
|  |  |  |  |  |  |
| VCA0731 |  | hypothetical protein | 2.89 | 7.40 | Hypothetical |
| VCA0279 |  | transcriptional regulator | 1.94 | 3.82 | Transcriptional regulator |
| VCA0684 |  | regulatory protein UhpC | 1.84 | 3.59 | Hexose-6 phosphate transport |
| VCA0338 |  | hypothetical protein | 1.67 | 3.18 | Hypothetical protein |
| VC1096 |  | hypothetical protein | 1.61 | 3.06 | Hypothetical protein |
| VC1418 |  | hypothetical protein | 1.51 | 2.84 | Hypothetical protein |
| VC1461 | *cep* | colonization factor | 1.37 | 2.58 | SOS regulon/ UV resistance |
| VC2367 |  | hypothetical protein | 1.28 | 2.44 | Hypothetical protein |
| VC2561 |  | uroporphyrin-III C-methyltransferase | 1.13 | 2.18 | Biosynthesis of cofactors/ porphyrin biosynthetic process |
| VCA0470 |  | acetyltransferase | 1.11 | 2.16 | Metabolic process |
| VCA0022 |  | glutathione S-transferase | 1.08 | 2.12 | T6SS |
| VCA0402 |  | acetyltransferase | 1.07 | 2.10 | Metabolic process |
| VC2618 |  | bifunctional N-succinyldiaminopimelate-aminotransferase/acetylornithine transaminase protein | 1.02 | 2.03 | Acetylornithine aminotransferase |
| VCA0789 |  | hypothetical protein | 0.99 | 1.99 | Hypothetical protein |
| VC0161 | *Ilvy* | DNA-binding transcriptional regulator IlvY | 0.92 | 1.89 | Transcriptional regulator/ branched amino acid regulon |
| VC2303 |  | hypothetical protein | 0.89 | 1.86 | Hypothetical protein |
| VCA0588 |  | peptide ABC transporter ATP-binding protein | 0.88 | 1.84 | Transport |
| VC2693 | *cpxA* | two-component sensor protein | 0.84 | 1.79 | Signal transduction histidine kinase |
| VCA0593 | *PggH* | phosphoguanylyl guanylate hydrolase | 0.81 | 1.75 | Signal transduction/ c-di-GMP levels |
| VC1069 |  | glyceraldehyde-3-phosphate dehydrogenase | 0.80 | 1.74 | Metabolism |
| VC2154 |  | hypothetical protein | 0.79 | 1.73 | Hypothetical protein |
| VCA0581 |  | hypothetical protein | 0.71 | 1.64 | Hypothetical protein |
| VC0749 |  | scaffold protein | 0.68 | 1.60 | Biosynthesis of cofactors/ iron-sulfur cluster |
| VC2076 |  | hypothetical protein | 0.67 | 1.59 | Hypothetical protein |
| VC2535 |  | peptidase PmbA | 0.67 | 1.59 | Metalloprotease |
| VC1455 |  | transcriptional repressor RstR | 0.67 | 1.59 | Transcriptional regulation |
| VC0095 | *ubiC* | chorismate--pyruvate lyase | 0.66 | 1.58 | Metabolism/ ubiquinone biosynthesis |
| VC2444 |  | general secretion pathway protein B | 0.66 | 1.58 | Transport |
| VC2296 |  | penicillin binding proteins/beta lactamase transcription regulator BolA | 0.65 | 1.57 | Transcriptional regulator/ Stress response |
| VC0968 |  | cysteine synthase A | 0.65 | 1.57 | Amino acid biosynthesis |
| VC1857 |  | primosomal replication protein N`` | 0.65 | 1.57 |  |
| VC1070 |  | phosphatase | 0.64 | 1.56 | Signaling |
| VC1464 |  | transcriptional repressor RstR | 0.63 | 1.55 | Transcriptional regulation |
| VCA0514 |  | hypothetical protein | 0.62 | 1.54 | Hypothetical protein |
| VCA0837 |  | hemolysin | 0.62 | 1.54 |  |
| VC2692 | *cpxR* | transcriptional regulator CpxR | 0.61 | 1.53 | Transcriptional regulator/Evelope stress response |
| VC2142 | *flaB* | flagellin | 0.60 | 1.52 | Motility |
| VCA0954 | *cheV4* | chemotaxis protein CheV | 0.60 | 1.52 | Signal Trasnduction/ Chemotaxis |
| VC1012 |  | electron transport complex protein RsxE | 0.60 | 1.51 | Energy metabolism/ electron transport |
| VC1215 | *pgsA* | CDP-diacylglycerol--glycerol-3-phosphate 3-phosphatidyltransferase | 0.60 | 1.51 | Fatty acid and phospholipid metabolism/Biosynthesis |
| VC0275 |  | phosphoribosylamine--glycine ligase | 0.60 | 1.51 | Purine ribonucleotide biosynthesis |
| VCA0741 |  | hypothetical protein | 0.59 | 1.51 | conserved hypothetical protein |
| VC2686 |  | hypothetical protein | 0.59 | 1.50 | conserved hypothetical protein |
| VC0965 | *ptsI* | phosphoenolpyruvate-protein phosphotransferase | -0.58 | 0.67 | Signal transduction/ Sugar transport/PTS |
| VC2653 | *secB* | preprotein translocase subunit SecB | -0.59 | 0.66 | Transport/protein export |
| VC0528 |  | 2-C-methyl-D-erythritol 4-phosphate cytidylyltransferase | -0.60 | 0.66 | Biosynthetic process |
| VC2108 |  | erythronate-4-phosphate dehydrogenase | -0.60 | 0.66 | Metabolic process |
| VC0004 |  | inner membrane protein translocase component YidC | -0.60 | 0.66 | Protein and peptide secretion and trafficking] |
| VCA1075 | *crvA* | curvature regulator in *Vibrio* A | -0.61 | 0.65 | Cell shape/Peptidoglycan insertion |
| VC2402 |  | cell division protein FtsW | -0.62 | 0.65 | Cell division/ peptidoglycan biosynthesis |
| VC0312 |  | FMN reductase | -0.64 | 0.64 | Electron Transport |
| VC0225 |  | lipopolysaccharide biosynthesis protein | -0.64 | 0.64 | Biosynthesis/Metabolic process/LPS |
| VC2568 |  | FKBP-type peptidylprolyl isomerase | -0.64 | 0.64 | Protein folding. |
| VC0891 |  | exodeoxyribonuclease VII small subunit | -0.66 | 0.63 | Degradation of DNA |
| VC1977 |  | aminotransferase | -0.66 | 0.63 | Biosynthesis |
| VC2191 | *flgM* | flagellar hook-associated protein FlgK | -0.67 | 0.63 | Chemotaxis and motility |
| VC2195 |  | flagellar basal body rod protein FlgG | -0.68 | 0.63 | Chemotaxis and motility |
| VC2280 |  | hypothetical protein | -0.71 | 0.61 | Hypothetical protein |
| VC1900 | *fadR* | fatty acid metabolism regulator | -0.72 | 0.61 | Transcriptional regulator |
| VC2308 |  | 4-methyl-5(B-hydroxyethyl)-thiazole monophosphate biosynthesis protein | -0.72 | 0.61 | Biosynthesis |
| VC0681 |  | bifunctional riboflavin kinase/FMN adenyltransferase | -0.75 | 0.60 | Biosynthesis of Riboflavin, FMN, and FAD |
| VC0019 |  | valine--pyruvate transaminase | -0.75 | 0.59 | Biosynthesis |
| VC0103 |  | hypothetical protein | -0.76 | 0.59 | Enzymes of unknown specificity |
| VC2083 |  | zinc ABC transporter permease | -0.76 | 0.59 | Transport/ Zinc |
| VC1684 |  | peptide ABC transporter ATP-binding protein | -0.83 | 0.56 | Transport |
| VC2533 |  | phosphocarrier protein NPr | -0.97 | 0.51 | Signal transduction:PTS |
| VC2395 |  | hypothetical protein | -1.61 | 0.33 | conserved hypothetical protein |
| VC0383 |  | hypothetical protein | -1.80 | 0.29 | conserved hypothetical protein |
| VCA0524 |  | hypothetical protein | -2.02 | 0.25 | conserved hypothetical protein |

**Table S6: List of primers used in the study**

| **Gene** | **Primer sequences** | **Source** |
| --- | --- | --- |
| *vpsT* | F: ACCTCTTTCGCATCAGGACAACTG | (He et al., 2012) (19) |
|  | R: CCTTTGGCGCTGGAAATTACACCA |  |
| *vpsH* | F: TTCAGGCATACCCTTCTTCG | Primer3Plus software (Untergasser et al., 2007)(18) |
|  | R: AGCCAAAGCGTGTCTCAATC |  |
| *bfd* | F: GGTTGCCGAACAGGGTATTA | Primer3Plus software (Untergasser et al., 2007)(18) |
|  | R: GAGCAATCAGCGATTCTTCG |  |
| *dncV* | F: GGATGATGATGCTCGTGATG | Primer3Plus software (Untergasser et al., 2007)(18) |
|  | R: GGCATTGGCATATAGGTTCC |  |
| 16s | F: GTGTAGCGGTGAAATGCGTAGAG | (Bachmann et al., 2015)(20) |
|  | R: GCGTGGACTACCAGGGTATCTAAT |  |
| *rpoD* | F: AGGCAGTGGCTCACGACCCAT | (Lee et al., 2012) (21) |
|  | R: ATGCGACTTGGTGGATCCGTCA |  |

References:

1. Abuaita BH, Withey JH. 2009. Bicarbonate Induces Vibrio cholerae Virulence Gene Expression by Enhancing ToxT Activity. Infect Immun 77:4111–4120.

2. Iwanaga M, Kuyyakanond T. 1987. Large Production of Cholera Toxin by Vibrio cholerae 01 in Yeast Extract Peptone Water. J Clin Microbiol 25:2314–2316.

3. Aristoteli LP, Willcox MDP. 2003. Mucin Degradation Mechanisms by Distinct Pseudomonas aeruginosa Isolates In Vitro. Infect Immun 71:5565.

4. Morimoto Y, Namba K, Minamino T. 2017. Bacterial Intracellular Sodium Ion Measurement using CoroNa Green. Bio Protoc 7:e2092.

5. Charles RC, Kelly M, Tam JM, Akter A, Hossain M, Islam K, Biswas R, Kamruzzaman M, Chowdhury F, Khan AI, Leung DT, Weil A, Larocque RC, Bhuiyan TR, Rahman A, Mayo-Smith LM, Becker RL, Vyas JM, Faherty CS, Nickerson KP, Giffen S, Ritter AS, Waldor MK, Xu P, Kováč P, Calderwood SB, Kauffman RC, Wrammert J, Qadri F, Harris JB, Ryan ET. 2020. Humans Surviving Cholera Develop Antibodies against Vibrio cholerae O-Specific Polysaccharide That Inhibit Pathogen Motility. mBio 11:1–13.

6. Russell JB. 1987. A proposed mechanism of monensin action in inhibiting ruminal bacterial growth: effects on ion flux and protonmotive force. J Anim Sci 64:1519–1525.

7. Huczyński A, Janczak J, Łowicki D, Brzezinski B. 2012. Monensin A acid complexes as a model of electrogenic transport of sodium cation. Biochimica et Biophysica Acta (BBA) - Biomembranes 1818:2108–2119.

8. Nickerson KP, Llanos-Chea A, Ingano L, Serena G, Miranda-Ribera A, Perlman M, Lima R, Sztein MB, Fasano A, Senger S, Faherty CS. 2021. A Versatile Human Intestinal Organoid-Derived Epithelial Monolayer Model for the Study of Enteric Pathogens. Microbiol Spectr 9:e0000321.

9. Llanos-Chea A, Citorik RJ, Nickerson KP, Ingano L, Serena G, Senger S, Lu TK, Fasano A, Faherty CS. 2019. Bacteriophage therapy testing against Shigella flexneri in a novel human intestinal organoid-derived infection model. J Pediatr Gastroenterol Nutr 68:509–516.

10. Verma S, Prescott RA, Ingano L, Nickerson KP, Hill E, Faherty CS, Fasano A, Senger S, Cherayil BJ. 2020. The YrbE phospholipid transporter of Salmonella enterica serovar Typhi regulates the expression of flagellin and influences motility, adhesion and induction of epithelial inflammatory responses. Gut Microbes 11.

11. Nyström EEL, Martinez-Abad B, Arike L, Birchenough GMH, Nonnecke EB, Castillo PA, Svensson F, Bevins CL, Hansson GC, Johansson MEV. 2021. An intercrypt subpopulation of goblet cells is essential for colonic mucus barrier function. Science 372:eabb1590.

12. Hase CC, Thai LS, Boesman-Finkelstein M, Mar VL, Burnette WN, Kaslow HR, Stevens LA, Moss J, Finkelstein RA. 1994. Construction and characterization of recombinant Vibrio cholerae strains producing inactive cholera toxin analogs. Infect Immun 62:3051–3057.

13. Mandlik A, Livny J, Robins WP, Ritchie JM, Mekalanos JJ, Waldor MK. 2011. RNA-Seq-based monitoring of infection-linked changes in Vibrio cholerae gene expression. Cell Host Microbe 10:165–174.

14. Millet YA, Alvarez D, Ringgaard S, von Andrian UH, Davis BM, Waldor MK. 2014. Insights into Vibrio cholerae Intestinal Colonization from Monitoring Fluorescently Labeled Bacteria. PLoS Pathog 10:e1004405.

15. Cameron DE, Urbach JM, Mekalanos JJ. 2008. A defined transposon mutant library and its use in identifying motility genes in Vibrio cholerae. Proc Natl Acad Sci U S A 105:8736–8741.

16. Gardel CL, Mekalanos JJ. 1996. Alterations in Vibrio cholerae motility phenotypes correlate with changes in virulence factor expression. Infect Immun 64:2246–2255.

17. Baranova DE, Willsey GG, Levinson KJ, Smith C, Wade J, Mantis NJ. 2020. Transcriptional profiling of Vibrio cholerae O1 following exposure to human anti- lipopolysaccharide monoclonal antibodies. Pathog Dis 78:ftaa029.

18. Untergasser A, Nijveen H, Rao X, Bisseling T, Geurts R, Leunissen JAM. 2007. Primer3Plus, an enhanced web interface to Primer3. Nucleic Acids Res 35.

19. He H, Cooper JN, Mishra A, Raskin DM. 2012. Stringent response regulation of biofilm formation in vibrio cholerae. J Bacteriol 194:2962–2972.

20. Bachmann V, Kostiuk B, Unterweger D, Diaz-Satizabal L, Ogg S, Pukatzki S. 2015. Bile Salts Modulate the Mucin-Activated Type VI Secretion System of Pandemic Vibrio cholerae. PLoS Negl Trop Dis 9:e0004031.

21. Lee KM, Park Y, Bari W, Yoon MY, Go J, Kim SC, Lee H Il, Yoon SS. 2012. Activation of cholera toxin production by anaerobic respiration of trimethylamine N-oxide in Vibrio cholerae. Journal of Biological Chemistry 287:39742–39752.
