# Supplementary material for: Human antibody targeting Vibrio cholerae O1 O-specific polysaccharide induces an amotile hypovirulent bacterial phenotype: mechanism of protection against cholera
Source: mBio. 2025 Sep 12;16(10):e02235-25. doi: 10.1128/mbio.02235-25 (PMC12505965; doi:10.1128/mbio.02235-25)
Supplement: Legends — for supplemental figures, tables, and movies. [file mbio.02235-25-s0007.docx]

**Supplemental Figure Legends**

**Suppl Fig. S1:** Box violin plots of fold-change in expression of target gene in *V. cholerae* exposed to mucin-G1/mucin-B12 as compared to bacteria in LB medium. The number of biological replicates (n) is indicated below each graph.

**Suppl Fig. S2: Cyclic di-GMP levels in medium containing LBM. (A)** Fluorescence measurements of assay carried out in LBM medium indicating higher background fluorescence of LBM (arrow). Data representative of two experiments. Further analysis therefore was carried out in the absence of mucin to assess impact of antibody on c-di-GMP levels. **(B)** Measurement of c-di-GMP in C6706 and rough mutant strain in the presence of G1 or B12 indicating absence of induction in rough strain. Data representative of two experiments.

**Suppl Fig. S3: Schematic for generation of enteroid-derived epithelial monolayer model secreting mucus. (A)** Enteroids maintained in Matrigel (top, left) were trypsinized to obtain single cell suspension that was seeded onto Transwell^®^ inserts (top, right), cultured to confluence and differentiated. Post-differentiation, mucus containing mucin secreted by monolayer was allowed to accumulate as shown in (**B** (uninfected)) before colonization with *V. cholerae* C6706 expressing tdTomato. The monolayers were visualized **(B-C)** by capturing three-dimensional confocal images (60X, Nikon) following staining with 4',6-diamidino-2-phenylindole (DAPI, blue) and lectin wheat germ agglutinin (WGA, green). Bacteria on top of mucus/surface glycoproteins appear red, while those within the mucus/surface glycoprotein structure appear yellow (red+green) as seen in sliced view in Fig. 7A.

**Suppl Fig. S4: CT detection in supernatants of *V. cholerae* infected duodenum-derived monolayers in the presence of G1 or B12.** (**A**) GM1 ELISA for CT detection in culture supernatant of duodenum-derived monolayers upon infection with *V. cholerae* C6706 with and without mucus and 0.0125 µM of anti-OSP G1 or anti-Flg B12. Data representative of two experiments. (**B**) GM1 ELISA for CT detection in culture supernatant of duodenum-derived monolayers with mucus accumulation upon infection with *V. cholerae* C6706 (black bars) or rough mutant (fuchsia bars) in the absence or presence of 0.0125 µM of G1 or B12. Data representative of two experiments.

**Suppl movie SM1:** A swimming *V. cholerae* cell (red) with flagellum (green) experiences intermittent and partial arrest of flagellar rotation while exposed to 0.0025 µM anti-OSP antibody. Gentle fluid flow is applied to the left, demonstrating that the arrested cell is not attached to the surface. Playback in real time (20 fps).

**Suppl movie SM2:** A swimming *V. cholerae* cell (red) with flagellum (green) changes from a swimming state to an arrested state while exposed to 0.025 µM anti-OSP antibody. Gentle fluid flow is applied to the left, demonstrating that the arrested cells are not attached to the surface. Playback in real time (20 fps).

**Supplemental Table Legends:**

**Table S1.** List of bacterial strains used in this study.

**Table S2.** Partial list of *V. cholerae* C6706 genes whose expression was altered by the presence of mucin alone (LB vs LBM) indicating fold change of DEGs (differentially expressed genes) organized into functional category and gene product, if known.

**Table S3.** List of *V. cholerae* C6706 genes whose expression was altered in the presence of both concentrations of anti-OSP antibody G1 (0.0125 µM and 0.125 µM) in LBM compared to LB alone indicating the fold change of DEGs along with functional category and gene product, if known.

**Table S4.** List of 33 *V. cholerae* genes whose expression was altered in the presence of 0.0125 µM G1 in mucin compared to LB alone with fold change and associated biological function or pathway, if known, indicated.

**Table S5.** List of 69 *V. cholerae* genes whose expression was altered in the presence of 0.125 µM G1 in mucin compared to LB alone with fold change and associated biological function or pathway, if known, indicated.

**Table S6.** List of primers used in the study.
